# Nurturing Care Systems Underlying Early Childhood Food Insecurity in Brazil: A Causal Loop Diagram Approach

**DOI:** 10.1111/mcn.70142

**Published:** 2025-11-29

**Authors:** Gabriela Buccini, Krystyna A. Stave, Katherine Marçal, Sonia Isoyama Venancio, Muriel Bauermann Gubert, Rafael Pérez‐Escamilla

**Affiliations:** ^1^ Department of Social and Behavioral Health, School of Public Health University of Nevada Las Vegas Nevada USA; ^2^ School of Public Policy and Leadership University of Nevada Las Vegas Nevada USA; ^3^ School of Social Work Rutgers The State University of New Jersey New Jersey USA; ^4^ Ministry of Health Brasilia Federal District Brazil; ^5^ Department of Nutrition University of Brasilia Brasilia Federal District Brazil; ^6^ Department of Social and Behavioral Sciences, School of Public Health Yale University New Haven Connecticut USA

**Keywords:** causal loop diagram, early childhood development, food insecurity, system thinking

## Abstract

Experiencing food insecurity in early childhood is associated with adverse health and nutrition outcomes. About 66% of Brazilian households with children are food insecure; however, interventions targeting early childhood have fallen short in addressing food insecurity due to a lack of funding and multisectoral coordination combined with the COVID‐19 pandemic. Therefore, protecting children from food insecurity is a complex task in Brazil and requires innovative approaches. We hypothesize that applying a Nurturing Care Framework (NCF) lens and systems thinking tools can indicate pragmatic pathways to reduce early childhood food insecurity. To examine this hypothesis, we used a participatory group model‐building approach to integrate the knowledge of twelve Brazilian experts working in different sectors into a qualitative causal loop diagram (CLD) underlying the dynamics of food insecurity in early childhood. By analyzing the CLD, we aimed (1) to assess whether the Brazilian food insecurity system includes the NCF components and identify how these systems affect young children, and (2) to qualitatively explore feedback loops, pivotal variables (i.e., variables with the most immediate causes and/or immediate causal consequences), and leverage points (i.e., specific variables to intervene to produce a change in the overall system) to reduce food insecurity in early childhood. The integrated CLD outlines a structure with 28 variables assigned across the NCF components. A deeper qualitative analysis of the direct and indirect links identified how food insecurity is experienced by young children. This included a description of two feedback loops reinforcing childhood food insecurity, such as Financial Hardships and Emotional Distress Spiral. Food quality emerged as the pivotal variable with the most proximate causes and/or consequences related to early childhood food insecurity. Positive parenting practices and participation in daycare emerged as direct links to address early childhood food insecurity. Expanding access to nurturing care through national and local policies can enhance the resilience of the systems underlying early childhood food insecurity to disruptions such as the COVID‐19 pandemic.

## Summary

- Early childhood food insecurity is linked to adverse outcomes, yet traditional linear models often fail to capture its complex dynamics nature.
- A group model‐building engaged multisectoral experts to construct a qualitative causal loop diagram that mapped these complex dynamics.
- Analysis revealed that Brazil's food insecurity system embeds nurturing care components, with two reinforcing loops—Financial Hardship and Emotional Distress—intensifying insecurity. Food quality emerged as a pivotal variable, while positive parenting and daycare participation were key intervention points.
- Strengthening access to nurturing care through coordinated national and local policies can enhance system resilience to future disruptions.

## 1 Introduction

Food insecurity is characterized by the lack of consistent access to sufficient, safe, and nutritious food to meet one's dietary needs for an active and healthy life (Pérez‐Escamilla 2024; World Food Summit 1996). In 2023, 2.3 billion people worldwide were food insecure; additionally, over 3.2 billion people were unable to afford a healthy diet (Food and Agriculture Organization of the United Nations FAO 2023). Experiencing food insecurity in early childhood from pregnancy to age three is associated with negative health and nutrition outcomes such as low birth weight (Harper et al. 2023), birth defects (Carmichael et al. 2007), chronic malnutrition (Bronte‐Tinkew et al. 2007; Lye et al. 2023; Metallinos‐Katsaras et al. 2009; Moradi et al. 2019), infectious diseases (Gubert et al. 2016), and developmental delays (De Oliveira et al. 2021; de Oliveira et al. 2020; Gallegos et al. 2021). Evidence from systematic reviews documented at least two causal pathways through which food insecurity may impact early childhood outcomes (Buccini et al. 2024; de Oliveira et al. 2020; Myers 2020; Patriota et al. 2024; Pedroso et al. 2020; Perez‐Escamilla 2013). In the biological pathways, food insecurity influences the child's dietary intake, nutritional status, and neurological development. In the socio‐emotional pathways, food insecurity leads to emotional strain for both the child and caregiver, limiting opportunities for cognitive development at home (Buccini et al. 2024; de Oliveira et al. 2020; Myers 2020; Patriota et al. 2024; Pedroso et al. 2020; Perez‐Escamilla 2013). Often the association between food insecurity and early childhood outcomes are examined using linear models. Hence, these analyses do not capture the real‐world complex dynamics underlying the food insecurity systems, including household agency, food availability, access, and utilization and their impact on early childhood outcomes (Clapp et al. 2022). Additionally, these analyses often disregard intrinsic time delays, such as oscillation related to food stability and sustainability, which can undermine the long‐term effectiveness of food insecurity interventions that are critical during early childhood (Clapp et al. 2022).

Brazil, the largest country in Latin America and the Caribbean, with approximately 215.3 million inhabitants, showcases the importance of considering the complex systems underlying food insecurity in early childhood (Pérez‐Escamilla et al. 2024). Between 2004 and 2013, food insecurity prevalence progressively decreased from 34.9% to 22.6% in the country (IBGE 2014; Pérez‐Escamilla et al. 2024). In the early 2000s, strong civil society participation enabled political commitment to optimize nutrition‐focused (e.g., regulation of food prices) and social protection (e.g., conditional cash transfer) interventions (Pérez‐Escamilla et al. 2024; Salles‐Costa et al. 2022), which effectively removed the country from the World Food Program's Hunger Map in 2014. However, this improvement was not sustained. Multisectoral coordination of public policies aimed at reducing food insecurity was disrupted by political changes (Santos et al. 2021), and economic instabilities lead to high unemployment rates, which were exacerbated during the COVID‐19 pandemic (Ribeiro‐Silva et al. 2020; Santos et al. 2021). As a result, Brazil was back on the Hunger Map in 2021, with 58.7% of households experiencing levels of food insecurity (Penssan 2022; Pérez‐Escamilla et al. 2024). Households with children were disproportionately impacted, with 66.1% experiencing food insecurity, of which 18.1% experienced severe food insecurity (Penssan 2022). Therefore, Brazil is far behind the United Nations Sustainable Development Goals of ending food insecurity in all its forms by 2030 (United Nations n.d).

Home‐visiting interventions targeting early childhood in Brazil have fallen short in addressing food insecurity due to barriers at the programming and system levels (Buccini et al. 2021; Buccini et al. 2024; Dos Santos et al. 2023). Designing new interventions or sustaining existing ones to protect the approximately 10 million young children living in extreme poverty from food insecurity has been a complex problem for decision‐makers in Brazil (Martins et al. 2024). At the same time, the discouraging dynamics in food insecurity over time support the importance of finding ways to stabilize and increase the resilience of the systems to reduce food insecurity in early childhood. The Nurturing Care Framework (NCF) provides a roadmap consisting of five components (i.e., Good Health, Adequate Nutrition, Opportunities for Early Learning, Security and Safety, and Responsive Caregiving) to transform child rights into equitable actions to address threats to optimal early childhood development (Black et al. 2017; Britto et al. 2017; UNICEF, World Bank, and World Health Organization 2018). The NCF has been shown as an adequate approach for determining assets to build resilience to address food insecurity among underserved communities (Buccini et al. 2024). Additionally, a recent UNICEF report calls for a strong NCF approach that is fully operationalized to decrease early childhood food insecurity effectively (UNICEF n.d). Therefore, in this study, we hypothesize that applying an NCF lens combined with complex systems thinking tools can be an innovative approach to document pathways to reduce early childhood food insecurity in Brazil.

To examine this hypothesis, we used a participatory group model‐building (GMB) approach (Anastasiou et al. 2023; Andersen and Richardson 1997; Gerritsen et al. 2020; Hovmand 2013; Hovmand et al. 2012; Meadows 2008; Vennix 1996; Vennix et al. 1996) to integrate the knowledge of Brazilian experts into a qualitative causal loop diagram (CLD) of systems underlying the dynamics (Meadows 2008) of food insecurity in early childhood. A CLD is a system thinking tool used to show how variables influence each other within a dynamic system over time. Relationships in CLDs are usually qualitative and heuristic, indicating whether the influence between variables is positive or negative, rather than specifying statistical associations such as in a directed acyclic graph (DAG) commonly used by epidemiologists (Tennant et al. 2021). Therefore, CLDs can be used to create shared understanding and to investigate emergent behaviors within systems and systemic drivers of problems (Hovmand 2013).

CLDs have been used to understand food insecurity in global settings (Richards et al. 2021), including the United States (Fleischer et al. 2017; Metta et al. 2021), Ethiopia (Ayenew 2015), and Cuba (Ruiz et al. 2022). To our knowledge, no previous CLD has attempted to document the systems underlying food insecurity in early childhood in a large Latin American country with sharp inequities like Brazil. In this case, CLD can be used to identify mechanisms within food insecurity system including feedback loops (i.e., change in one variable feeds through causal links and eventually causes a change in the original variable), pivotal variables (i.e., variables with the most immediate causes and/or immediate causal consequences), and leverage points (i.e., specific variables to intervene to produce a change in the overall system). Insights from the CLD can be used to inform policies and actions to break undesired trends in food insecurity in early childhood. By analyzing a co‐created CLD, the objective of this study is twofold: (1) to assess whether the Brazilian food insecurity system includes the NCF components and identify how these systems affect young children, and (2) to qualitatively explore feedback loops, pivotal variables, and leverage points to reduce early childhood food insecurity.

## 2 Methods

### 2.1 Study Design

**Figure 1:**
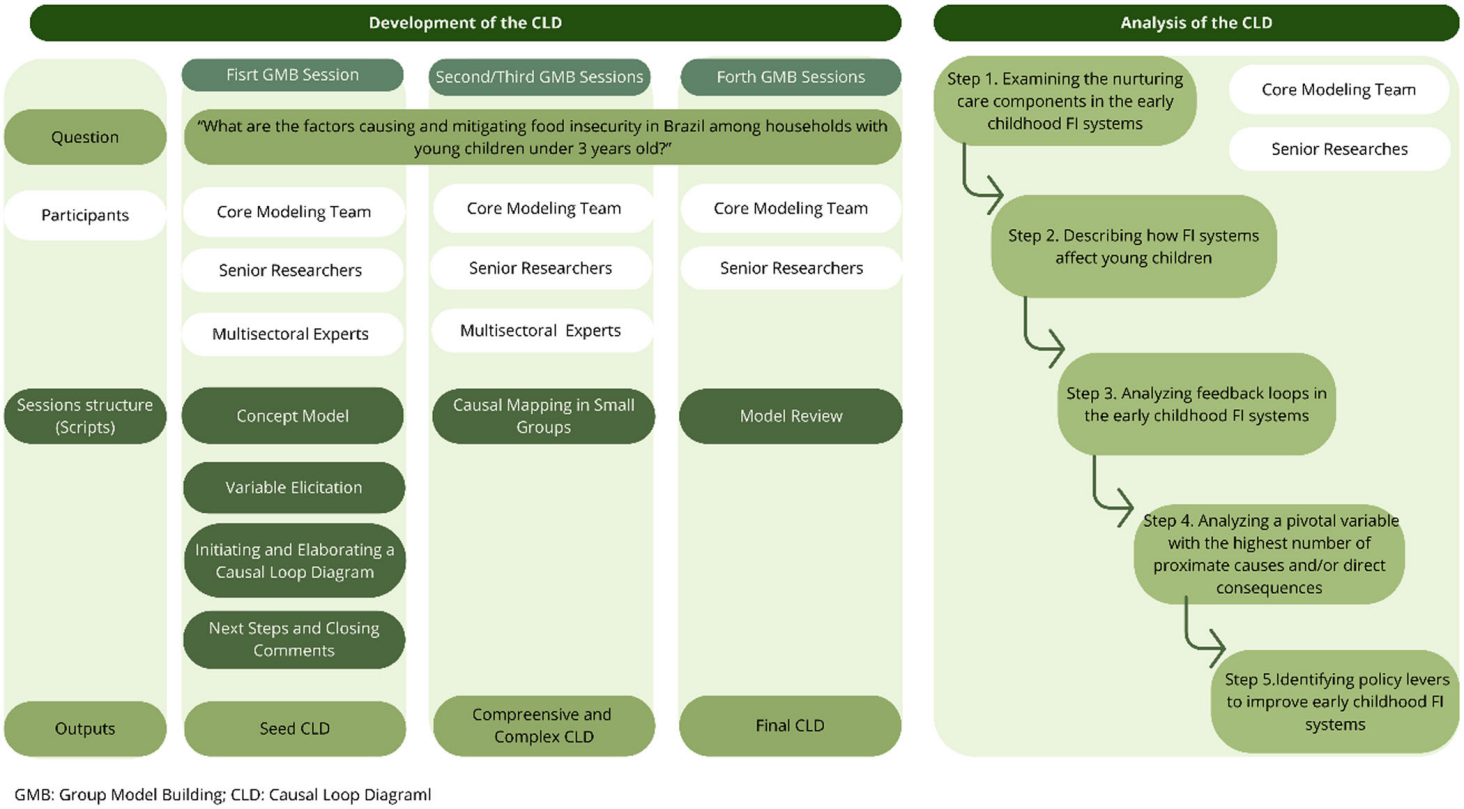
Steps to develop and analyze the causal loop diagram of early childhood food insecurity in Brazil.

This exploratory qualitative case study planned a group model‐building approach to codesign a CLD assessing whether the Brazilian system includes the NCF components, and to explore qualitatively feedback structures, pivotal variables, and leverage points to reduce early childhood food insecurity. In brief, our research team designed and conducted structured GMB sessions to elicit and organize participant knowledge of the problematic trend of early childhood food insecurity in Brazil. An overview of the methodological approach to develop the CLD is outlined in Figure 1.

This study received ethical approval from the Research Ethics Committee of the Health Institute of the São Paulo State Health Department (n. 3.320.733) and by the Institutional Review Board of the University of Nevada, Las Vegas (n. 1702327). Participants engaged in data collection activities from May 17, 2022 to March 25, 2023. All participants provided verbal consent after a description of the study's purpose and design. We followed the Consolidated Criteria for Reporting Qualitative Studies (COREQ) (Tong et al. 2007) to report this manuscript (Appendix 1).

### 2.2 Group Model‐Building Data Collection

#### 2.2.1 Research Team's Positionality

The “core modeling team” (GB, KM, KS) consisted of three researchers, who are faculty members at U.S. higher education institutions. One is an expert in maternal‐child public health nutrition (GB), and the other two are senior experts in system dynamics and participatory modeling (KM, KS). GB is a native Portuguese speaker and had a prior relationship with several participants, which facilitated the high attendance of participants in the GMB sessions. The research team consisted of the “core modeling team” and three additional senior researchers who engaged in the analysis of the CLD. Two of the senior researchers are faculty members at Brazilian higher education institutions (MG and SIV) and have experience in child health, program evaluation, and household food insecurity. SIV was a consultant for the Brazilian Ministry of Health at the time of the data collection. The third senior researcher (RPE) is a faculty member at a private U.S. higher education institution and has worked for over three decades in Brazil in the areas of maternal, infant, and young child feeding, as well as household food and water insecurity. In recent years (2019–2023), the research team interviewed 171 teams at national, state, and municipal‐level implementing early childhood initiatives in Brazil as well as 95 families engaging in these early childhood initiatives (Buccini et al. 2021; Buccini et al. 2024). The lessons learned from interviewing the families experiencing food insecurity were considered in the design of the CLD (Buccini et al. 2025; Dos Santos et al. 2023).

#### 2.2.2 Participants

The twelve participants in four GMB sessions consisted of the three members of the “core modeling team” (GB, KM, KS), the three senior researchers (MG, SIV, RPE), and six specialists working in various sectors in Brazil. Multisectoral specialists were selected through a convenience sampling approach ensuring balanced representation across different sectors and ministries that have programs related to early childhood and/or food security. The specialists were selected by the core modeling team because of their expertise and high‐level positions in various sectors of the Brazilian ministries. This included representatives from the Food and Nutrition Coordination (*n* = 3) and the Breastfeeding Coordination (*n* = 1) within the Ministry of Health and representatives from the Early Childhood Home‐Visiting Program *Criança Feliz* (*n* = 1) and the *Bolsa Familia* Conditional Cash Transfer Coordination (*n* = 1) within the Ministry of Social Development. The specialists held at least a bachelor's degree and had experience working with families of young children at risk for food insecurity. In their positions within ministries, they conducted technical visits to states and municipalities and listening sessions with families enrolled in public programs. Several had recent frontline engagement with families that included children under 3 years of age and who were experiencing food insecurity, allowing these perspectives to be indirectly represented in the modeling sessions. Each specialist contributed with in‐depth knowledge related to their sector, which helped to develop a comprehensive and integrated CLD. All participants invited to the GMB sessions agreed to participate.

#### 2.2.3 Sessions Structure

We selected scripts (i.e., standardized protocols for small group exercises that guide participants through various activities) available through the Scriptapedia, which is a handbook for developing structure GMB sessions (Hovmand et al. 2011; Luna‐Reyes et al. 2006; Vennix 1995). In this study, we selected GMB scripts based on three primary nature of group task: presentation (i.e., activities intended to educate or update participants on specific skills, concepts, or insights related to system dynamics modeling), divergent (i.e., activities that aim to produce a wide array of different ideas and interpretations from a group), and convergent (i.e., tasks that focus on consolidating or organizing ideas, interpretations, and information into thematic clusters or categories). The selection of the scripts was based on the primary group task and facilitation strategies to implement the activities virtually. As recommended by the Scriptapedia handbook, a single scripted activity should focus on only one primary task to keep the activity clear and focused. Thus, when a session required both a divergent and a convergent activity, we selected two separate scripts (Hovmand et al. n.d). The selection of the scripts was supervised by one of the researchers who is an expert in system dynamics (KM). An example of GBM script used can be found in Appendix 2 and at the Scriptapedia handbook (Hovmand et al. n.d). Table 1 summarizes the scripts used per sessions, their goals, facilitation techniques used, and key insights.

**Table 1:** Group model building (GMB) scripts description.

GMB session	Who?	When?	Scripts	Script description^a^				
				Goal	Primary nature of group task	Input	Facilitation evaluation criteria	Key insights
#1	Core modeling team, senior experts in maternal‐child health, Multisectoral specialists	February 2022	(a) “Concept Model”	To introduce the process of modeling and symbolism of a model to participants	Presentation	None	Participants are talkative, wanting to tell the modeler how the model is wrong and can be improved.	Familiarity with stock and flow diagrams and causal links
								Understanding that maps can be quantified and simulated
							Participants can use the symbols and terms of system dynamics to express their own experience and knowledge of systems.	Understanding that models can be created for the group's problem(s)
								Understanding that the model is owned by the group and can be repeatedly modified and improved
			(b) “Variable elicitation”	To facilitate consensus‐based group discussion about the model problem and boundaries	Divergent	None	Identification of key variables and stocks	Prioritized list of variables
			(c) “Initiating and Elaborating a Causal Loop Diagram”	To get an initial idea of central concepts and their relationships	Convergent	List of prioritized variables	Improvement in quality of communication, insight, consensus on the problem, and commitment with regard to actions	Consensus on dynamic hypothesis
							Improved causal loop diagram	Initial general CLD
			(d) “Next steps and closing comments”	To identify next steps and close the group model building session	Convergent	Deliverables from session (e.g., printouts of model)	Everyone in the room knows the next steps	
#2	Core modeling team, senior experts in maternal‐child health	May 2022	“Causal Mapping in Small Groups”	To clarify and advance the development of the CLD by building inclusive conversations within subgroups	Divergent	Reference Model Graph over time charts A list of primary and secondary variables	Improvement in quality of communication, insight, consensus on the problem, and commitment with regard to causal loop diagrams	Interim output/product: increased consensus on dynamic hypothesis, or a possible structural explanation for observed behavior within sub‐groups
							Participants understand the overall concepts of building causal loop diagrams and generate a set of causal loop diagrams that reflect group consensus on the problem and tend to converge to a final group causal loop diagram	Deliverable: sub‐group causal loop diagrams which would be used as forming sub groups' dynamic hypothesis and establishing group consensus of a convergent group model.
#3	Core modeling team, Multisectoral specialists	June 2022						
							Inclusive discussions within sub‐groups and joint group conversations	
#4	Core modeling team, Senior experts in maternal‐child health	November–February 2023	“Model Review”	To summarize the successive versions of the CLD, clarify fuzzy ideas, capture additional information about model structure, and elicit feedback from participants on the final CLD	Convergent	Diagram of model	A revised causal loop diagram that is based on an initial discussion	List of main feedback loops and dynamics identified
							A shared understanding of the changes in the model and insights that have emerged	List of insights gained from the link circle exercise and subsequent model

a Full description of scripts can be found at Scriptapedia website.

Four scripts were used to guide the GMB session #1: (a) The “Concept Model” script was used at the start of a GMB session to introduce the process of modeling a CLD to participants (Scriptapedia n.d.‐b); (b) The “Variable Elicitation” script was used to facilitate consensus‐based group discussion about the model problem and boundaries early in the modeling process (Scriptapedia n.d.‐f); (c) The “Initiating and Elaborating a Causal Loop Diagram” script was used to provide an initial idea of central concepts and their relationships at the beginning of the project (Scriptapedia n.d.‐c); and (d) The “Next steps and closing comments” script was used to identify the next steps and close the group model‐building session (Scriptapedia n.d.‐e).

The follow‐up GMB sessions #2 to #4 were scheduled to be short in length and at times that were convenient for participants. In GMB sessions #2 and #3, the “Causal Mapping in Small Groups” script was used to gather divergent perspectives from subgroups of participants to advance the development of the CLD (see Table 1) (Scriptapedia n.d.‐a). In the GMB session #4, the “Model Review” script was used to summarize the evidence gathered into a final CLD, clarify unclear ideas, capture additional information, and elicit feedback from participants on the final CLD (see Table 1) (Scriptapedia n.d.‐d).

#### 2.2.4 Facilitation Techniques

All GMB sessions were conducted virtually via Zoom and recorded. Effective facilitation is critical in GMB, particularly in an online format where non‐verbal cues are harder to detect. The virtual sessions were conducted by GB who has +10 years of experience conducting virtual and in‐person participatory activities to engage participants. We used strategies to manage challenges like online fatigue (i.e. short sessions about 1 h long and continued follow up sessions), and ensure active participation (i.e., prompt questions directly to quieter participants, and asking for examples among participants). Scripts guide procedures to engage all participants in generating ideas for broader discussion. The initial GMB session was conducted in English with attendance of 12 participants and follow up GMB sessions were conducted with small groups in Portuguese. Only the initial GMB session was conducted in English to allow the participation of the entire research team, and while the language was not a barrier for participants to engage in the session, the option of adopting the participants native language (i.e., Portuguese) in the follow up sessions aimed to ensure deeper discussions and facilitate continued engagement during the online interactions. To capture non‐verbal input, in the initial section, two members of the core modeling team (KM, KS) had the role of observing and following up with prompts if a participant was quieter or deviating from the initial prompt to develop the CLD. In the follow up sessions, a Portuguese speaker researcher assistant took that role. The facilitator kept the environment positive. If there were disagreements during the discussion, the facilitator helped the group to reach a consensus by asking clarifying questions to gain more insight on each participant's perspective. Consensus was reached when the participants agreed on either adding a factor, renaming, or removing it.

#### 2.2.5 Building the CLD

In the GMB session #1, participants were introduced to the project objectives and the GMB methodology as well as to a seed CLD structure developed from the literature review. The core modeling team reviewed the Food and Agriculture Organization of the United Nations (FAO) Global Strategic Framework for Food Security and Nutrition (Food and Agriculture Organization of the United Nations [FAO] n.d)., systematic reviews on early childhood food insecurity (de Oliveira et al. 2020; Perez‐Escamilla 2013), and technical reports addressing early childhood food insecurity in Brazil. These readings informed a “Seed CLD Structure” used at to jump‐start the process. Then, the core modeling team posed the following question: “What are the factors causing and mitigating food insecurity in Brazil among households with children under 3 years old?” Participants were then encouraged to brainstorm factors driving early childhood food insecurity and discuss the links between these factors. Factors could either corroborate the factors outlined in the seed concept model or be a new addition expanding the model. The session concluded upon reaching saturation, which occurred when subjects were consistently reiterated, no novel ideas emerged from participants, and the participants felt that the seed model had been sufficiently developed (Appendix 3, see figure “Seed CLD Structure”).

In GMB sessions #2 and #3 with small groups of participants, the seed CLD structure developed in the first session was refined. During these sessions, emphasis was placed on evaluating the accuracy, clarity, and comprehensiveness of the diagram. Participants identified missing factors or links and proposed modifications based on their expertise in the whole system as well as specific parts of the system. These iterative refinements resulted in a more comprehensive and complex model (Appendix 3, see figure “Complex CLD Structure”).

The GMB session #4 consisted of multiple iterations with senior consultants in child health and nutrition in Brazil (MG, SIV) for final feedback. For example, due to the high number of factors in the model, the senior consultants suggested that the core modeling team group factors to simplify the visualization. Based on the literature review previously done to prepare for the GMB, the core modeling team grouped the factors representing similar concepts or themes into high‐level concepts to generate new variables. For example, “Quality of Food in Household” grouped factors qualifying the food available within the household, including dimensions of adequacy (meeting nutrient needs), balance (variety of food groups), safety (free from contaminants), age‐appropriateness (catering to specific age groups, such as breastfeeding), and cultural relevance (respecting dietary traditions). The grouping of factors within variables was refined through iterations with one senior researcher in maternal‐child health and nutrition in Brazil (MG). This process generated a streamlined visualization of the variables and links in the CLD. This improved version of the CLD was presented one more time to the senior researchers in maternal‐child health and nutrition (MG, SIV, RPE) for final feedback, ending the four‐session iterations. The final CLD is a shared representation of the system structure generating the trend in early childhood food insecurity in Brazil, based on the knowledge of specialists in multiple sectors involved in addressing food insecurity in Brazil.

### 2.3 Data Analysis

The final CLD produced during data collection as well as the transcription of the GMB sessions were the data sources analyzed in this study. Session recordings were transcribed by a professional company, and a research assistant checked the transcriptions for accuracy before the data were used for analysis. These materials were used to:

- Assess whether the Brazilian food insecurity systems includes the NCF components and identify how food insecurity systems affect young children;
- Explore qualitatively feedback structures, pivotal variables, and leverage points to reduce food insecurity in early childhood.

#### 2.3.1 Elements of a CLD

A CLD is a visual representation of the links between variables in a system. Arrows indicate links between two linked variables. The plus or minus symbol indicates whether the two linked variables change in the same (+) or opposite (−) directions. A segment in a CLD showing A → ( + ) B, for example, should be read as “a change in A causes a change in B in the same direction.”

A feedback loop or causal loop is a set of relationships where a change in one variable feeds through several causal links and eventually causes a change in the original variable (Lannon 2012). The feedback loops are either reinforcing (R)—when a change in any variable in the loop eventually amplifies the direction of the initial change in the same variable; or balancing (B)—when a change in any variable in the loop ultimately results in an opposite change in the same variable (Ballard et al. 2021; Pagoni and Patroklos 2019).

Finally, in a CLD, leverage points refer to specific variables to intervene in the system to produce a change in the overall system behavior. The most effective interventions for long‐term change are often those within feedback loops (Meadows (1999).

#### 2.3.2 Qualitative Analysis Approach

The research team consisting of six members who constructed the CLD including the core modeling team and senior researchers conducted a qualitative analysis of the links in the final CLD produced (Appendix 4). Variables were classified as endogenous (i.e., internal to the system and influenced by other variables within the system) and exogenous (i.e., external factors that affect the system but are not affected by variables within the system). Then, the research team followed a data analysis approach that included the five analytical steps described below. In the qualitative coding process, for intercoder reliability, a consensus meeting between the two members of the research team was used as standard approach (Cole 2024). Given the interpretive nature of this coding process, achieving inter‐coder reliability was important to demonstrate the clarity and communicability of the coding system, as well as to enhance the theory emerging from the data (Cole 2024). Any disagreements in the qualitative coding process were solved by consensus; additionally, a third member of the research team reviewed it for accuracy.

##### 2.3.2.1 Step 1. Examining the Nurturing Care Components in the Food Insecurity System

A deductive qualitative coding (i.e., pre‐defined categories based on existing framework) was used to identify the nurturing care components underlying the food insecurity system in Brazil. Two members of the research team independently coded variables in the final CLD according to one of the five components of the NCF following pre‐determined definitions: Good Health (i.e., considers the health care of both caregivers and children), Adequate Nutrition (i.e., considers the nutrition of both caregivers and children), Safety and Security (i.e., considers safe and secure environments for children and their families), Opportunities for Early Learning (i.e., considers opportunities for children to interact with a person, place, or object in their environment), and Responsive Caregiving (i.e., considers the ability of caregivers to provide choices, use a comforting and accepting voice, and offer physical affection, as well as respond to the children's signals in a timely and appropriate manner) (UNICEF, World Bank, & World Health Organization 2018). Any disagreements in the qualitative coding process was solved by consensus based on the standard approach for intercoder reliability as explained above; additionally, a third member of the research team reviewed it for accuracy. At the end of this step, an operational description of each variable was developed and the number of variables by NCF components was computed.

##### 2.3.2.2 Step 2. Describing How Food Insecurity Systems Affect Young Children

A deductive qualitative coding was used to assigned variables within the levels of the socio‐ecological framework (McLeroy et al. 1988). Two members of the research team independently coded variables in the final CLD according to one of the socio‐ecological levels following pre‐determined definitions: community level (the broader social context in which families and children are embedded), household level (the immediate family environment where the child resides), caregiver level (the immediate caregiver or parent responsible for the well‐being of household and the young child), and young child level (the individual child and their development). Any disagreements in the qualitative coding process were solved by consensus based on the standard approach for intercoder reliability as explained above; additionally, a third member of the research team reviewed it for accuracy. At the end of this step, we computed the frequency and percentage of variables by socio‐ecological levels; then, we described the variables directly linked to the young child's food insecurity separately from the household unit of analysis.

##### 2.3.2.3 Step 3. Analyzing Feedback Loops Affecting Food Insecurity in Early Childhood

Four members of the research team used an inductive qualitative coding (i.e., themes to emerge from the data) to identify feedback loops reinforcing food insecurity among households with young children. The coding process identified several feedback loops. To streamline the data analysis, only feedback loops that met the following criteria were analyzed: (a) if they uncover root causes of food insecurity in early childhood or (b) if they facilitate understanding or unintended consequences of leverage points for intervention. At the end of this step, the four members of the research team named the included feedback loops representing the overarching theme of the set of causal links within the loop. Then, a brief description of each feedback loop was provided.

##### 2.3.2.4 Step 4. Analyzing the Pivotal Variable With the Most Proximate Causes and/or Direct Consequences

Four members of the research team used an inductive qualitative coding to identify pivotal variables (i.e. with the most immediate causes and/or immediate causal consequences) within the CLD. As previously explained, these variables are relevant because changing one of their causal links might be insufficient if other causal links are not also addressed. To streamline the data analysis, only the pivotal variable with the highest number of causes and consequences was included in the data analysis. At the end of this step, a brief description of the pivotal variable was provided.

##### 2.3.2.5 Step 5. Identifying Policy or Program Leverages to Improve Food Security in Early Childhood

Four members of the research team used an inductive qualitative coding to identify leverage points that could move the system toward action for reducing levels of early childhood food insecurity. At the end of this step, arrows were added to the CLD indicating each potential policy or program leverages and identifying whether the intervention targets household or community/national level. Then, a brief description of each policy or program leverage was provided.

##### 2.3.2.6 Ethics statement

This study received ethical approval from the Research Ethics Committee of the Health Institute of the São Paulo State Health Department (n. 3.320.733) and by the Institutional Review Board of the University of Nevada, Las Vegas (n. 1702327).

## 3 Results

**Figure 2:**
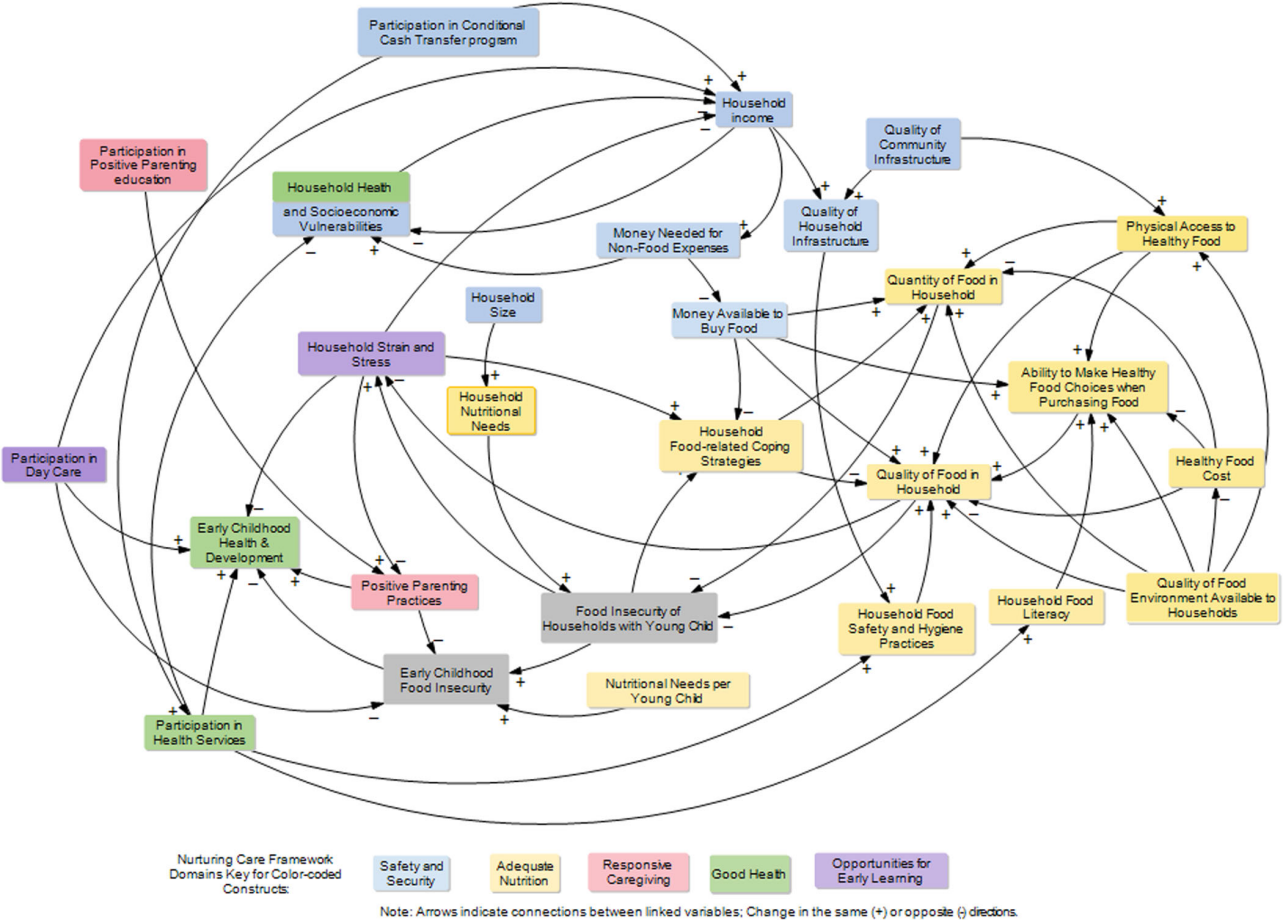
Causal map of the systems underlying early childhood food insecurity.

The final CLD developed through the participatory GMB consisted of 28 variables driving early childhood food insecurity in Brazil (Figure 2). A total of 23 variables were endogenous to the system influenced by other variables within the system; and, 5 variables were exogenous or external factors not affected by the system (Table 2). In the next section, the names of the variables are presented in *italics*.

**Table 2:** Operational definition of the variables outlined in the causal loop diagram of food insecurity among households with young children across the nurturing care and socio‐ecological frameworks.

Nurturing care framework	Variable name and definition	Type of variable	Socio‐ecological framework			
			Young child	Caregiver	Household	Community
Outcomes	***Food Insecurity of Households with Young Child*** refers to the limited or uncertain availability of nutritionally adequate and safe foods, or the limited or uncertain ability to acquire such foods in socially acceptable ways, within households with children under three years of age. This can be due to unavailability of food and/or lack of resources to obtain food.	Endogenous			*	
	***Early Childhood Food insecurity*** refers to the lack of availability and provision of nutritious, adequate, and safe foods for children under three years old. This condition can result from a lack of financial resources, inadequate access to food, or other factors that disrupt a household's ability to provide a consistent and healthy diet for their young children.	Endogenous	*			
Good Health	***Early Childhood Health and Development*** encompasses the holistic growth of children, including their physical, cognitive, linguistic, social, and emotional development. It also considers individual needs such as child temperament, illness, and disabilities, as these factors influence their experience with food security. The period from pregnancy to three years old is critical because early experiences shape brain architecture and influence future success in school, work, and life. To support optimal development, children need nurturing care, health and nutrition, family and community support, and enabling environments tailored to their individual needs.	Endogenous	*			
	***Participation in Health services*** refers to the active engagement of individuals and families in the Brazilian public healthcare system, known as the *Unified Health System (SUS)*. It includes seeking preventive care, such as vaccinations and check‐ups, as well as utilizing curative services when needed. A key component of participation in healthcare services is enrollment in the *Family Health Strategy (Estratégia Saúde da Família, ESF)*, a primary care model that provides comprehensive and continuous care to individuals and families in their communities. Participation in the ESF and the broader SUS system is a right for all Brazilian citizens and residents, regardless of their socioeconomic status.	Endogenous				*
	***Household Health Vulnerability*** refers to the susceptibility of a household to health risks and challenges due to various factors that can affect their overall well‐being. These factors include inadequate access to healthcare services, poor living conditions, exposure to environmental hazards, and lack of resources for preventive care and treatment. Additionally, individual health conditions, chronic illnesses, and limited health literacy within the household further contribute to this vulnerability.	Endogenous			*	
**Responsive Caregiving**	***Positive Parenting Practices*** involve creating a loving and stimulating home environment where parents show attention and affection, respond to the child's needs, communicate and play with them, and offer age‐appropriate learning and exploration opportunities. Additionally, they establish predictable routines and clear boundaries with positive discipline, avoiding harsh punishment, while prioritizing the physical and mental well‐being of the entire family. Also include a responsive feeding approach that focuses on recognizing and responding to a child's hunger and fullness cues, while creating a nurturing environment. This approach fosters a positive feeding relationship, encourages self‐regulation of food intake, and supports the child's overall development, ultimately promoting healthy eating habits and a positive relationship with food.	Endogenous		*		
	**Participating in Positive Parenting Education** such as through *Programa Criança Feliz (PCF)*, provided within the framework of the Brazilian Social Assistance System (SUAS) helps caregivers learn how to support early childhood development comprehensively aiming strengthen family bonds, promoting early learning and child development, and improve well‐being outcomes by empowering caregivers with knowledge and skills to better respond to their children's needs.	Exogenous		*		
**Opportunity for Early Learning**	***Household Strain and Stress*** refers to the emotional, psychological, and physical strain experienced by individuals or families, encompassing tension, pressure, and disruptions within household relationships due to financial difficulties, work‐related stress, health issues, parenting responsibilities, marital conflicts, or external pressures.	Exogenous			*	
	***Participation in Day Care*** refers to enrollment in Public Day Care Centers in Brazil. Day Care is part of the Brazilian Education System. These educational institutions offer free care and age‐appropriate education for children from 0 to 3 years old. Enrollment is not mandatory, and coverage is approximately 50%. Public Day Care Centers also provide nutritious meals free of charge while the child is under their care.	Exogenous	*			
**Adequate Nutrition**	***Quality of Food in Household*** refers to the nutritional value of available food within the household, encompassing the types and proportions of foods and food groups available. It emphasizes the importance of adequacy (meeting nutrient needs), balance (variety of food groups), safety (free from contaminants), age‐appropriateness (catering to specific age groups, such as breastfeeding), and cultural relevance (respecting dietary traditions), thereby promoting optimal growth, development, and overall health.	Endogenous			*	
	***Quantity of Food in Household*** refers to the amount and portion sizes of available food within the household necessary to meet the energy and nutrient needs of all household members within a specific timeframe (e.g., daily or per meal). Adequate quantity is crucial to support optimal growth, development, and overall health	Endogenous			*	
	***Ability to Make Healthy Food Choices when Purchasing Food*** involves the ability to make food choices that promote a balanced, nutritious, and beneficial diet for people within the household, emphasizing in‐natura or minimally processed foods and limiting the purchase of ultra‐processed foods. This ability is influenced by various factors, including food literacy, access and affordability of healthy foods, cultural and social influences, food marketing and advertising, personal preferences and habits, time constraints and convenience, as well as emotional and psychological factors.	Endogenous			*	
	***Household Food Literacy*** encompasses the knowledge and skills necessary to make informed decisions about food and health. It involves understanding nutrition information, such as food labels and dietary guidelines, and applying that knowledge to choose healthy foods and prepare nutritious meals. It also includes making conscious, sustainable, and culturally appropriate food purchases. Additionally, food literacy involves the ability to critically evaluate nutrition information from various sources and make evidence‐based decisions.	Endogenous			*	
	***Physical Access to Healthy Food*** refers to the availability and proximity of healthy food sources in the home or community, such as home gardens, supermarkets, food retail stores, farmers’ markets, and small local groceries. It includes factors like the distance to food sellers or producers and the availability of transportation to access these locations.				*	∙
	***Healthy Food Cost*** refers to the price that the final consumer pays for healthy food items.	Endogenous				*
	***Quality of Food Environment Available to Households*** encompasses the physical, economic, political, and socio‐cultural factors that influence the availability, accessibility, affordability, and desirability of food within a community. A high‐quality food environment supports healthy eating by providing easy access to a wide variety of nutritious, safe, pesticide‐free, and sustainable foods. It also limits the availability and promotion of ultra‐processed foods. A low‐quality food environment, on the other hand, is characterized by limited access to healthy foods and an abundance of unhealthy options. This can manifest as food deserts, areas with limited access to affordable and healthy food, often due to a lack of grocery stores or farmers' markets, and food swamps, areas saturated with fast food restaurants and convenience stores selling mostly ultra‐processed foods. Marketing and advertising also play a significant role in shaping the food environment. Aggressive marketing tactics for unhealthy foods, particularly those targeting children, can heavily influence food choices and preferences. A high‐quality food environment restricts these practices, empowering individuals to make informed decisions.	Exogenous				*
	***Household Food Safety and Hygiene Practices*** refers to the practices and conditions within a household that prevent food contamination and foodborne illness. It encompasses proper food handling, storage, preparation, and cooking techniques, as well as maintaining a clean and sanitary environment	Endogenous			*	
	***Household Food‐related Coping Strategies*** are adaptive measures families employ to ensure access to food and protect children and vulnerable people within the household from the adverse effects of food insecurity or limited resources. Common coping methods include reducing meal sizes, relying on cheaper options, rationing, using leftovers creatively, seeking food assistance, or growing food. Alternatively, they can be nonfood based, including borrowing money or food, selling assets, taking on additional work, reducing spending on other necessities, relocating, or seeking government assistance.	Endogenous			*	
	***Household Nutritional Needs*** refers to the collective nutritional requirements of all individuals residing within a household. These needs are determined by the specific dietary requirements of each person, based on their individual characteristics such as age, sex, activity level, physiological state (e.g., pregnancy, lactation), and health status. Household nutritional needs are not altered by external factors related to the household itself, such as socioeconomic status or food security. Each person's nutritional needs remain unique and independent of the household context.	Endogenous			*	
	***Nutritional Needs per Young Child*** refers to the specific nutritional requirements of both the mother and the developing child during pregnancy and the first three years of life, which are not altered by external factors related to the household itself, such as socioeconomic status or food security. These needs encompass a wide range of macro and micronutrients essential for optimal growth, development, and long‐term health. These needs are not static but evolve throughout the 1,000‐day period, with specific nutrients being particularly important at different stages.	Endogenous	*			
**Safety and Security**	***Household Income*** refers to the total amount of money earned by all members of a household within a specific period, typically a month or a year. It includes various sources of income, such as wages, salaries, self‐employment earnings, investments and pensions.	Endogenous			*	
	***Participation in Conditional Cash Transfer Program*** refers to the active enrollment and engagement of eligible families in the Brazilian government's conditional cash transfer program, *Bolsa Família (PBF)*, part of the Brazilian Social Assistance Policy. Eligible families, typically those living in extreme poverty, receive monthly financial assistance on the condition that they fulfill certain requirements related to health and education.	Exogenous			*	
	***Household Size*** refers to the total number of individuals living together in a single dwelling and sharing living arrangements, regardless of their age, relationship, or economic contribution to the household.	Exogenous			*	
	***Money Needed for Nonfood Expenses*** refers to the financial resources allocated by a household to cover essential living expenses beyond food. It includes expenditures on housing (mortgage or rent), utilities (electricity, gas, water), transportation (car payments, fuel, public transportation), healthcare (insurance premiums, medical expenses), education (tuition, school supplies), clothing, and other essential goods and services.	Endogenous			*	
	***Money Available to Buy Food*** refers to the financial resources a household has specifically allocated or available for acquiring food to meet the dietary and nutritional needs of all its members. This includes money that can be used to purchase groceries, meals at restaurants, or ingredients for home‐cooked meals.	Endogenous			*	
	***Household Socioeconomic Vulnerability*** encompasses a combination of factors that collectively disadvantage a household compared to others, impacting their well‐being and access to resources. These factors include socioeconomic characteristics such as income level, education, employment status, and housing conditions. Additionally, demographic characteristics such as race, gender, age, and household structure contribute to this vulnerability.	Endogenous			*	
	***Quality of Household Infrastructure*** encompasses both the external and internal components of households that contribute to a safe, healthy, and functional living environment. External infrastructure refers to access to essential community services like clean water supply, sewage systems, and electricity, while internal infrastructure focuses on in‐home amenities crucial for food preparation and storage. This includes a functional kitchen with adequate counter space and storage, reliable plumbing and electricity, appliances like refrigerators and stoves, and proper ventilation.	Endogenous			*	
	***Quality of Community Infrastructure*** includes the availability and access to essential services such as electricity, clean water supply, and sewage systems. It also encompasses well‐maintained transportation networks for both food and people, ensuring the efficient delivery of fresh produce and healthy food, as well as enabling individuals to easily reach grocery stores and markets.	Exogenous				*

### 3.1 Examining Nurturing Care Components

In the CLD, the two variables considered outcomes were *food insecurity in households with young children* and *early childhood food insecurity*. Out of the remaining 26 variables, 10 were assigned to Adequate Nutrition, nine to Security and Safety, three to Good Health, and two each to Responsive Caregiving, and Opportunities for Early Learning (Table 2). This classification confirmed the Brazilian food insecurity system includes nurturing care components.

### 3.2 Describing How Food Insecurity Systems Affect Young Children

Through the perspective of the socio‐ecological levels, of the 28 variables in the CLD, four variables were assigned to the community level, including *quality of food environment available to households*, 18 to the household level, such as *quality of household infrastructure*, *household income*, *money needed for nonfood expenses*, *household health, and socioeconomic vulnerabilities*, two to the caregiver level, including *positive parenting practices*, and *participating in positive parenting education*, and four to the young child level (Table 2).

**Figure 3:**
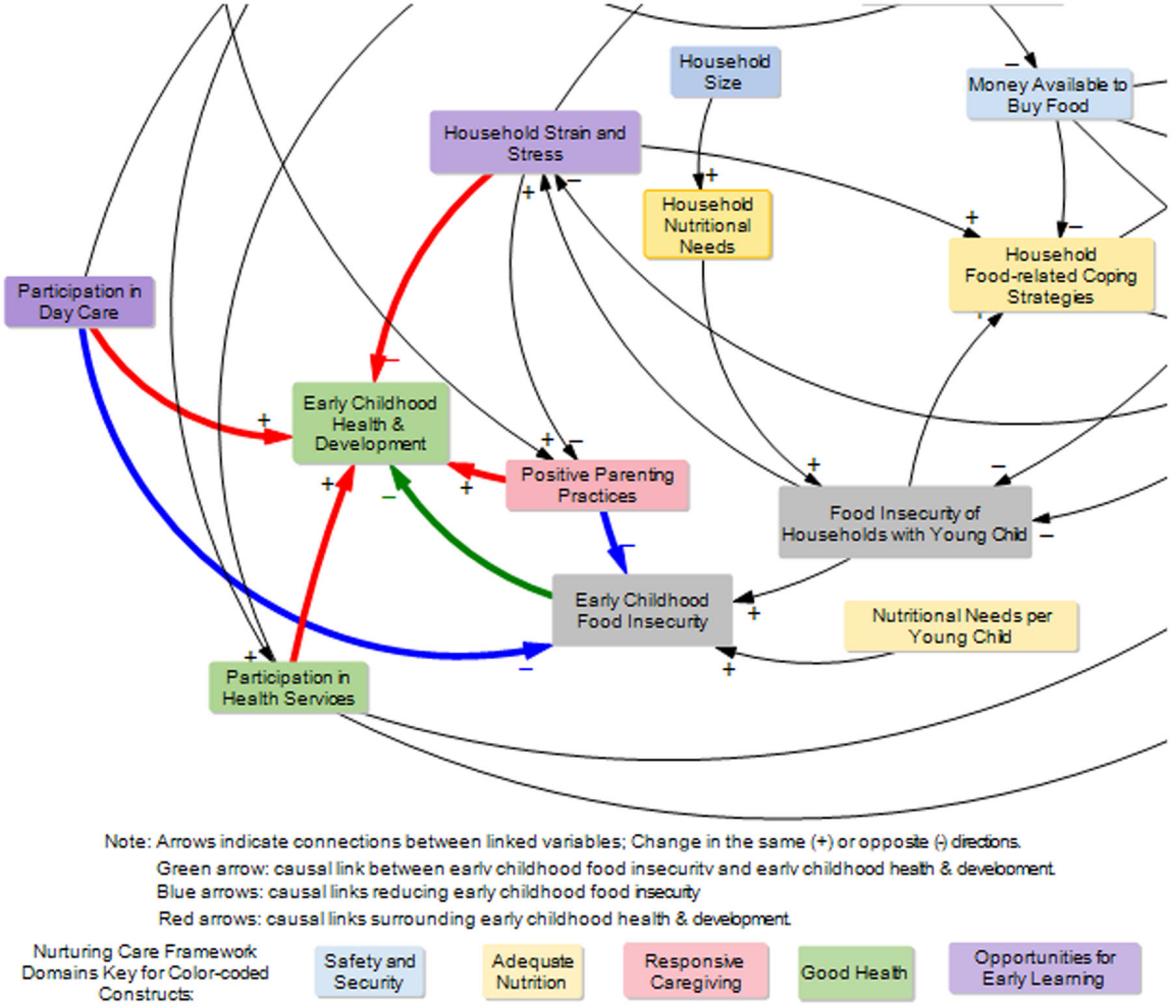
Situating young children in the causal map of the system underlying early childhood food insecurity.

We identified two causal links reducing *early childhood food insecurity*; one causal link is through *positive parenting practices*, and the second link is through *participation in daycare* (Figure 3, blue arrows). The presence of *early childhood food insecurity* decreases *early childhood health and development* (Figure 3, green arrow). In addition, causal links surrounding early childhood food insecurity and early childhood health and development were identified. For instance, *early childhood health & development* is a function of *positive parenting practices* (+), *participation in daycare* (+), *participation in healthcare* (+), and *household strain and stress* (−) (Figure 3, blue arrows).

### 3.3 Analyzing the Main Feedback Loops

**Figure 4:**
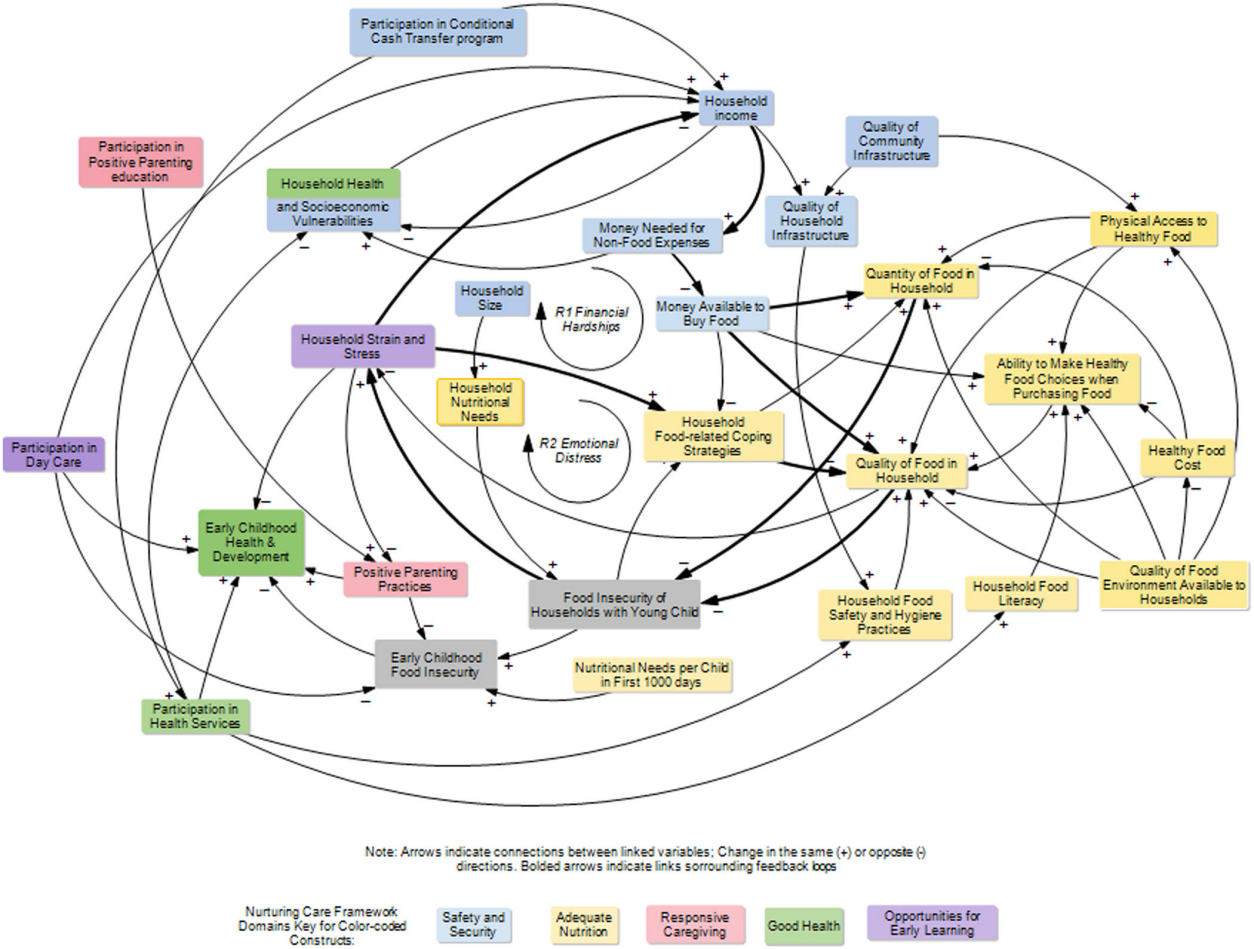
Main feedback loops in the causal map of the system underlying early childhood food insecurity.

The CLD contains several feedback loops. In particular, two reinforcing loops consisting of variables across three NCF components (Security and Safety, Adequate Nutrition, and Opportunities for Early Learning) emerged from our analysis. These two loops confirmed the most straightforward links between *household income*, *money available to buy food*, the *quantity and nutritional quality of food* that can be acquired by the household, and their food insecurity level. The first loop connecting *household income* with *food insecurity of households with young children* is shown as R1 Financial Hardships in Figure 4. The loop shows that when income falls, less money is available to buy food, and food insecurity among households with young children rises. Rising food insecurity among households with young children leads to increased *household strain and stress*, which feeds back to further decrease household income. This shows how a decrease in income or increase in stress can lead to a reinforcing downward spiral that compounds food insecurity among households with young children. It also shows the potential for an increase in income to reverse food insecurity among households with young children. A boost in income would increase the amount and quality of food, decrease food insecurity among households with young children, decrease household stress, and further boost income.

The second loop connecting *household strain and stress* with *food insecurity of households with young children* is depicted as R2 Emotional Distress Spiral in Figure 4. The loop begins when food insecurity among households with young children increases, it increases the levels of household stress, which in turn pushes households to turn to food‐related coping strategies. These food insecurity coping strategies among households with young children at first sacrifice food quality, which may maintain calories but can reduce nutritional quality and contribute to increasing food insecurity. Again, rising food insecurity among households with young children creates more household stress and leads to further food‐related coping practices.

### 3.4 Analyzing a Pivotal Variable

**Figure 5:**
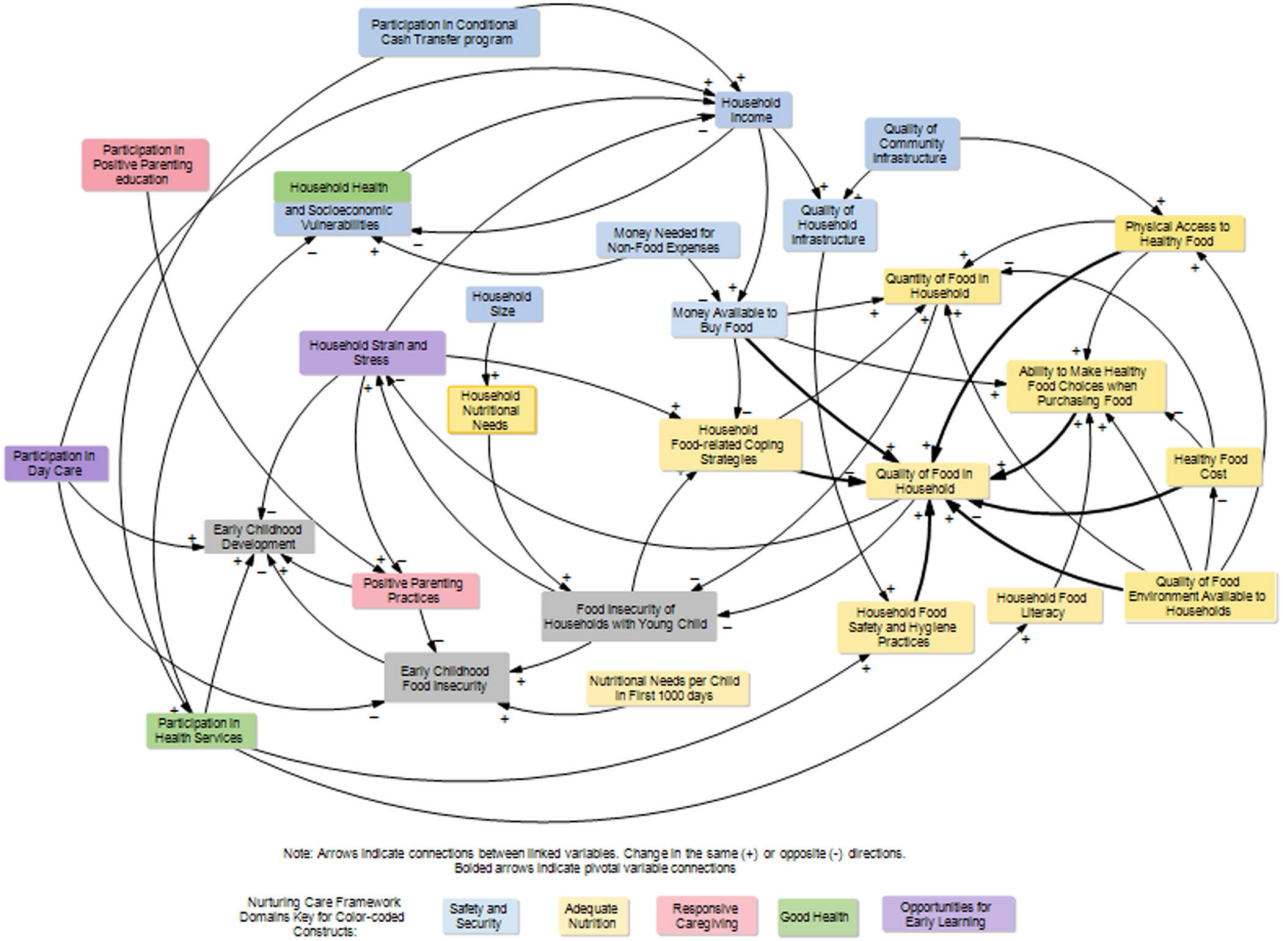
Pivotal variable with a higher number of inputs in the causal map of the system underlying early childhood food insecurity.

While there are multiple key variables in the diagram, *quality of food in households* emerged as a highly relevant variable with a particularly high number of causes. In essence, a household needs more than money to buy quality food as the food needs to be available, accessible, and reasonably priced. Once the food is obtained, the household also must have the ability to prepare and store it safely. Figure 5 shows the seven proximate variables (six from the NCF Adequate Nutrition domain) leading to food quality change: *money available to buy food* (the only variable from the Safety and Security NCF domain), *physical access to healthy food, ability to make healthy food choices when purchasing food, cost of healthy food, quality of food environment available to households, household food safety and hygiene practices, and household food‐related coping strategies* (Figure 5).

### 3.5 Identifying Program/Policy Leverages

**Figure 6:**
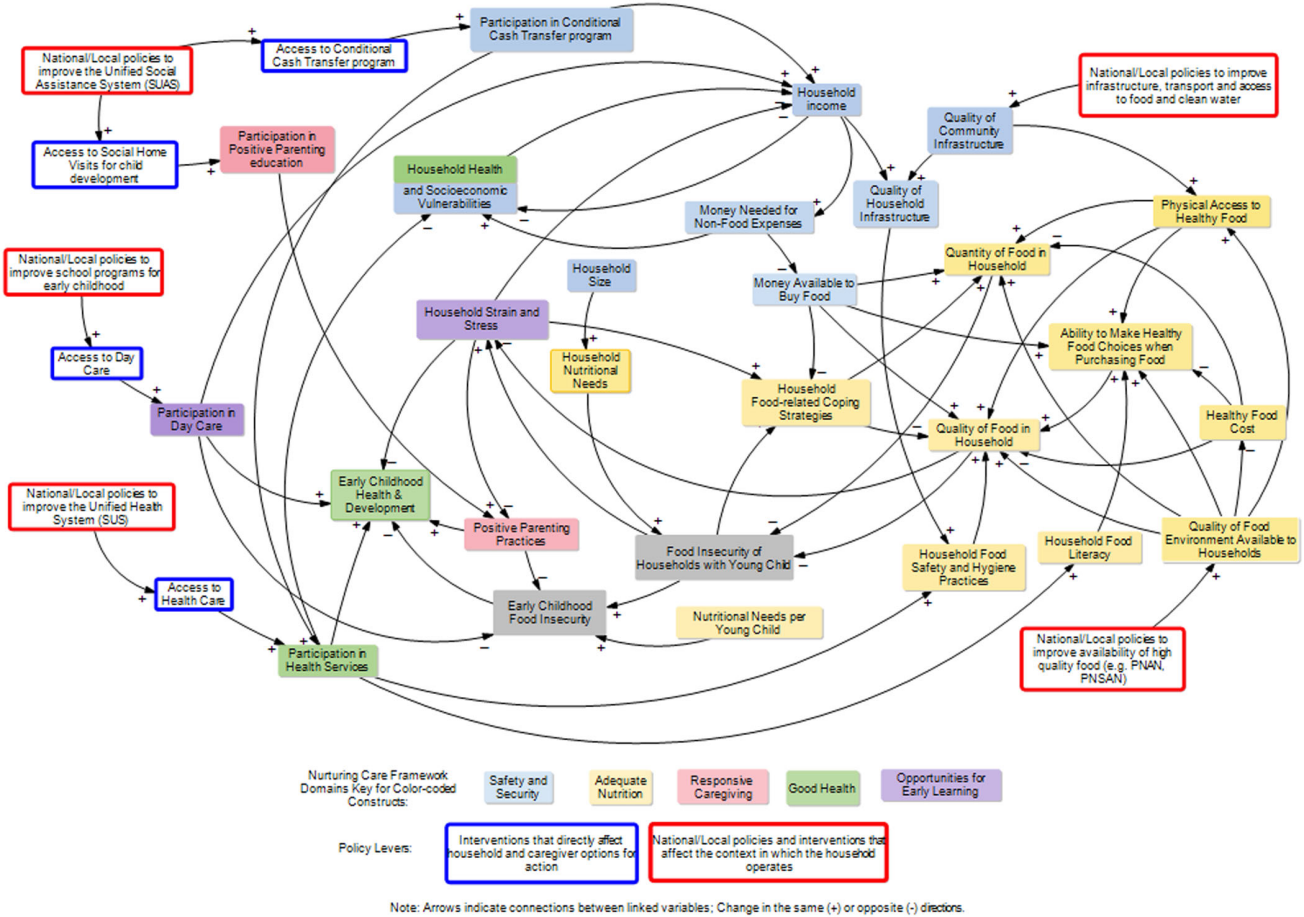
Leverages to improve early childhood food insecurity in Brazil.

Two tiers of policy levers were identified. The first tier included improving access to interventions that could modify some of the indirect links of food insecurity among households with young children (see blue outlined boxes in Figure 6). For instance, improving caregiver knowledge and skills can be achieved relatively quickly through targeted social programs. The second tier included interventions at the community/national level that could improve the context in which households operate (see red outlined boxes in Figure 6). For example, some more distal causal links, such as *quality of community infrastructure* and *quality of food environment available to households*, are more difficult to change in the short term and usually require national/local policy‐driven structural interventions over a longer term.

## 4 Discussion

Our study used a participatory approach to gather and integrate the knowledge of multisectoral specialists involved in addressing food insecurity in Brazil. This knowledge was integrated into a CLD outlining the complex food insecurity system structure among households with young children. When interpreting our findings, because of the systems approach inherent in our methodology, the links in the CLD should not be seen as statistical causal inferences; instead, they reflect and recognize the complex reciprocal processes among the systems, services, families, and the young child. Through qualitative analysis of the CLD, our research team confirmed the hypothesis that the Brazilian system does include the NCF components. In our study, the global NCF was a useful theory of change for modeling the complex food insecurity systems structure impacting early childhood health & development. This corroborates with a previous study where the NCF was deemed a well‐suited theory of change to understand how to address food insecurity in young children living in underserved communities (Buccini et al. 2024). Additionally, the NCF assisted in identifying policy levers to increase household agency in accessing a safety net program as a way to address early childhood food insecurity. Household agency refers to the capacity of individuals and households to make informed decisions about their own food systems, and to act on those decisions with dignity. This concept goes beyond simply having access to food and emphasizes the importance of choice, empowerment, and participation in the food system (Clapp et al. 2022). Prior evidence established household ability to access services is a critical component for advancing equitable livelihoods in sustainable food systems (Clapp et al. 2022). Therefore, strengthening the nurturing care components of early childhood food systems can enhance the sustainability and resilience through disturbances such as the COVID‐19.

Classifying variables across NCF components provided insights into the nurturing components underlying early childhood food insecurity. Surprisingly, separating out young children from the household unit was critical to identify variables assigned to both Responsive Caregiving (e.g. *responsive parenting practices*) and Opportunities for Early Learning (e.g. *participation in daycare programming)* components, which illuminated causal links to reduce early childhood food insecurity. Unsurprisingly, the majority of the variables were assigned to the Adequate Nutrition and the Security and Safety components, which is consistent with factors that have previously been associated with food insecurity among households with young children (Bortolini et al. 2015; Lignani et al. 2020; Poblacion et al. 2016). Our analysis of the CLD confirmed that food insecurity among households with young children is directly determined by the amount and quality of food available to households for a given level of food demand. Both food quantity and quality are variables historically associated with food insecurity among households with young children globally (Mulatu et al. 2024; Williams et al. 2024) and in Brazil (Bortolini et al. 2015; Carneiro et al. 2024). It is worth mentioning that the NCF components underlying the food insecurity systems were weakened between 2014 and 2024 due to political decisions that impacted funding and multisectoral coordination, leading to worsening food insecurity which was exacerbated during the COVID‐19 pandemic, particularly for households with young children (de Oliveira et al. 2020; Penssan 2022; Ribeiro‐Silva et al. 2020; Santos et al. 2021). Thus, several insights emerged from closer analysis of specific areas in the CLD.

First, separating out young children from the household unit clarified two direct links that support decreases in early childhood food insecurity. One was through positive parenting practices, including responsive feeding. A study in Brazil confirmed that responsive feeding practices were associated with food security, as reflected by optimal infant and young children feeding practices (Coleta et al. 2022). For example, the prevalence of exclusive breastfeeding was lower in the context of food insecurity (Buccini et al. 2024). It is important to recognize that responsive feeding in the context of early childhood food insecurity can be shaped by the child's temperament, disability, and level of autonomy around food and eating. For example, children who are temperamentally difficult, ill, or have a disability may present extra challenges, as well as children experiencing neophobia or seeking more autonomy during mealtimes. Therefore, the interpretation of these links in our CLD should consider the child's agency and role in the feeding process. The second link was through participation in daycare programs where young children generally receive supplemental nutrition, responsive care, and early stimulation, which in turn can buffer the effects of household food insecurity and related pressures and stress on young children. The suspension of daycare programs during the COVID‐19 pandemic highlighted the critical safety net role of such programs in feeding young children (Bauer et al. 2021; Pérez‐Escamilla et al. 2020). To our knowledge, how much daycare programs can be a safety net for early childhood food insecurity has not been well studied, particularly in Brazil. However, the multisectoral specialists engaged in developing the CLD noted this safety net feature of daycare programs in the Brazilian context. Understanding these links, we recommend equity‐focused protocols (Buccini et al. 2025) for optimizing or integrating interventions targeting the well‐being of children under 3 years old.

A second set of insights emerged from examining feedback loops. At the household level, the Financial Hardships and Emotional Distress spiral were the main feedback loops reinforcing early childhood food insecurity. The Financial Hardships feedback loop indicated that money for food is a necessary, but not sufficient, condition for healthy eating and food security, as previously reported (Lignani et al. 2020; Salles‐Costa et al. 2022). The number of residents and housing‐related costs were variables reported by the participants and integrated into our CLD, which have been found in prior studies, that pose an additional burden on the household budget that can worsen food insecurity. The Emotional Distress spiral feedback loop highlights the role of food‐related coping strategies in managing household strain and stress and nutrition‐specific variables. This feedback loop has been previously reported (Leung et al. 2022). The literature describes caregiver emotional distress as an emotional response to the stress related to the pressures faced by caregivers of balancing financial commitments, stigma for utilizing public assistance resources (e.g., a food pantry), and shame and guilt over their inability to adequately provide for their children (Jandaghian‐Bidgoli et al. 2024; Leung et al. 2022). Similar to the structure described by the participants from their experience in the Brazilian context, food‐related coping strategies such as food rationing and stretching are used as an attempt to protect young children from food insecurity (Chaudhuri et al. 2021). Coping strategies associated with an emotional response to stress include disengagement with daily activities, anger/violence, substance abuse, self‐harm, and social isolation (Chaudhuri et al. 2021; Jandaghian‐Bidgoli et al. 2024). While each feedback loop individually impacts the household as described above; both loops can occur simultaneously and amplify the negative impact on early childhood health and development. Evidence from the Family Stress Model shows that family economic hardship triggers financial pressure, which is defined as a persistent state of financial worry and strain, including experiencing food insecurity. This then affects parental mental health, increasing psychological distress, which in turn impacts parenting quality, leading to negative parenting behaviors such as increased conflict or harshness—all of which ultimately negatively impact early childhood health and development (Conger et al. 2002; Justice et al. 2025).

The *Quality of Food* in the household was identified as a pivotal variable due to the high number of direct and indirect links to proximate variables causing food quality to change. According to the Brazilian Dietary Guidelines, food quality is a complex variable defined as the ability to provide a varied, balanced diet based on fresh or minimally processed foods that is culturally appropriate, safe, tasty, and promotes sustainability (Brasil, Ministério da Saúde, Secretaria de Atenção Primária à Saúde, Departamento de Promoção da Saúde 2019). Prior research in Brazil has found that food insecurity simultaneously increases consumption of ultra‐processed foods, decreases fresh food choices, and lowers the quantity of any type of food, eventually leading to hunger (Bortolini et al. 2015; Poblacion et al. 2016). Thus, understanding these links is important because addressing one pathway without considering the others can lead to unexpected outcomes. For example, increasing *money available to buy food* in a household might not be sufficient to improve the *quality of food* in the household because other factors, such as inability to acquire high quality food due to lack of *physical access to healthy food*, which can obstruct or limit the effect of having money available to buy it. Another limiting factor is the geographical inequities in household infrastructure, such as lack of access to clean water, electricity, and stoves, which are common in Brazil and may impact food security (IBGE 2018; Penssan 2022). On the other hand, there is the possibility of the child or family dislike the high‐quality food, which would prevent them to buy it. Therefore, increasing nutrition and food literacy through participation in health services may enable healthier choices (Silva et al. 2023).

**Figure 7:**
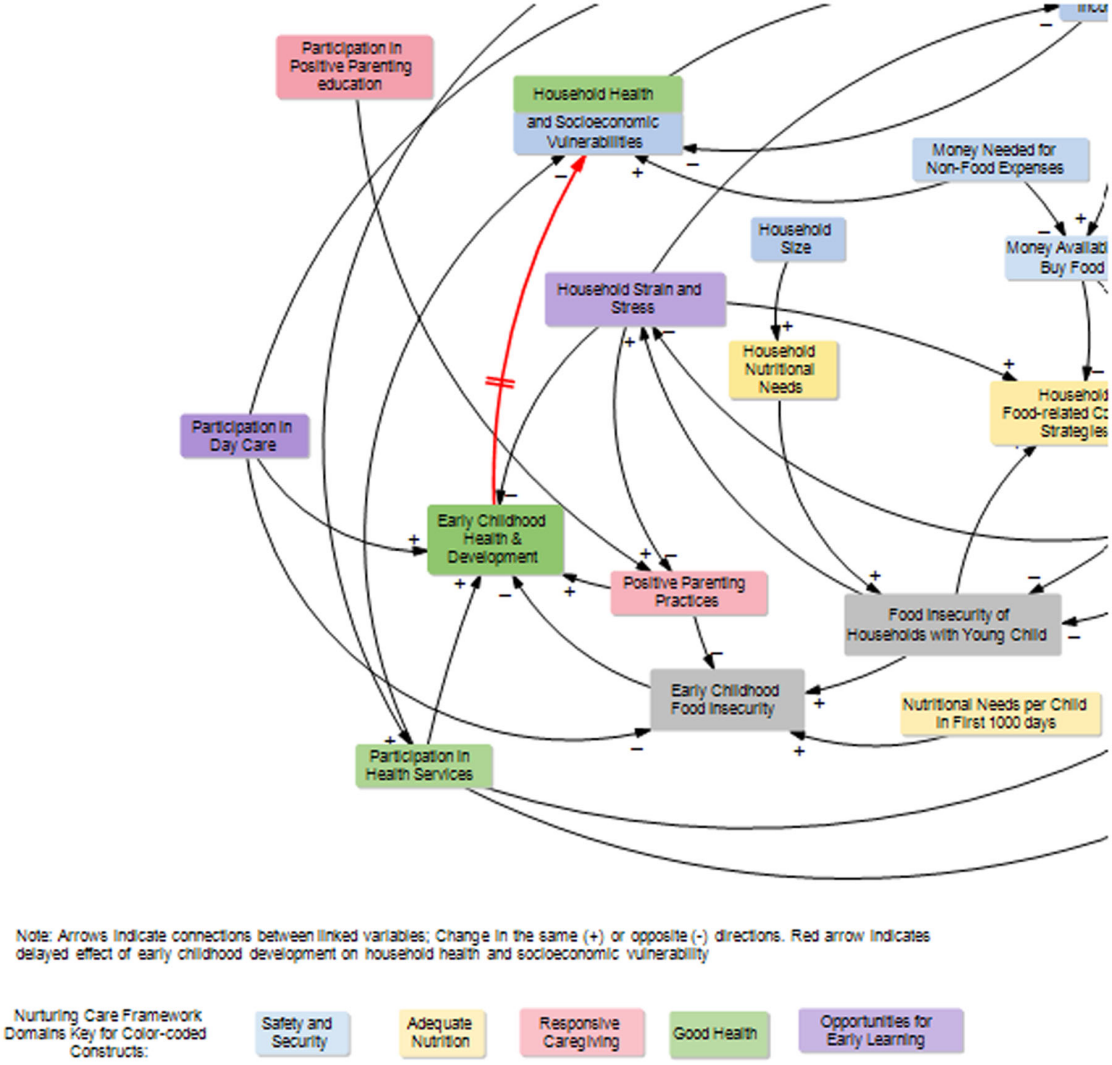
Delayed effect of early childhood development on household health and socioeconomic vulnerabilities.

Two tiers of policy levers were identified. One tier focused on the household level, and the other tier focused on the community and regional level. Some policy interventions amplify the effectiveness of some variables, which in turn accelerate or promote improvements in early childhood food insecurity. Examples include interventions like *participation in conditional cash transfer program*, which subsequently increase *household income* and should increase the *money available to buy food*. In Brazil, the Bolsa Família conditional cash transfer has successfully reduced levels of food insecurity among households with young children(Martins et al. 2024; Neves et al. 2022). However, one intervention is insufficient to prevent or mitigate early childhood food insecurity. Thus, it is important also to consider leverage points where variables may limit the effectiveness of other variables if they are weak or non‐existent. For example, if households have *money to buy food* but do not have *food and nutrition literacy*, their ability to make *healthy food choices when purchasing food* is limited. Similarly, if a household does not have a food preparation area (e.g., kitchen) with infrastructure to cook food or keep it from spoiling, their ability to choose healthy food may be limited even if they have enough money or the food and nutrition literacy needed. Hence, the system has several leverage points where interventions would be necessary to reduce system‐driven resistance to accessing, preparing, and consuming healthy and nutritious foods. For example, household pressures leading to stress were identified as a key resistor in the system and may counteract other variables in the system. Emotional distress makes it difficult for caregivers to use their parenting skills, for example, to practice positive parenting, which in turn has a negative effect on both early childhood food insecurity and early childhood health and development (Buccini et al. 2024; de Oliveira et al. 2020; Myers 2020; Patriota et al. 2024; Pedroso et al. 2020; Perez‐Escamilla 2013). The long‐term value of investing in a combination of interventions to mitigate early childhood food insecurity can enable better early childhood health and development outcomes and ultimately lower disparities in health and socioeconomic in adulthood as illustrated in Figure 7. Although Brazilian public policies for promoting early childhood are comprehensive and follow the NCF, there are still gaps in service access and challenges in the integration of the interventions across sectors to protect young children from food insecurity (Buccini et al. 2025; Carmo and Guizardi 2017).

Our study has strengths to consider in interpreting and generalizing the findings presented. First, we recruited diverse participants that collectively had the breadth and depth of knowledge needed to inform the CLD study and we had a very low dropout rate. The participatory process used to develop the CLD provided a platform for individual participants to contribute their knowledge and connect it with the contributions from other sectors. The process of expanding the causal representation, eliciting divergent views, and facilitating convergence around common variables helped key informants see the depth and breadth of the food insecurity system in Brazil. In this way, the contributions and interactions of the group participants stitched together the comprehensive CLD of the system. The system thinking approach promoted collaboration between our public health research team and system dynamics experts using a common language during the CLD analysis. At the end of the process, all participants expressed a high degree of confidence that the resulting CLD made the whole system visible and faithfully represents the pathways underlying food insecurity in Brazil. A strength of the study was the potential to foster interactions between multisectoral specialists and researchers to develop a common understanding of a wide‐reaching and complex system of early childhood food insecurity and its different drivers at different levels of the socio‐ecological framework. The dynamic process of expanding, diverging, and converging helped multisectoral specialists to collectively explain in great depth and breadth the food security systems surrounding households with young children in Brazil. The interdisciplinary nature of the system thinking approach allowed our public health research team to collaborate with systems dynamics experts to establish a shared understanding and common language during the data analysis.

Nevertheless, several challenges limit the interpretation of our results. First, although the feasibility and validity of participatory online approaches are well‐documented (Chavez‐Ugalde et al. 2022; Halliday et al. 2021), the online format of our GMB sessions may have influenced the process due to online fatigue and the limited ability to track participants’ non‐verbal cues. In addition, building the CLD was time‐consuming, which meant that some participants could not join in all the GMB sessions, limiting the collective discussion. To address these limitations, our research team employed several facilitation techniques described in the methods sections, including scheduling short meetings at convenient times for participants, conducting activities in Portuguese, their native language, and sharing materials before and after meetings for familiarization. These techniques aim to reduce participant fatigue and keep them engaged throughout the GMB sessions. After each session, the facilitator asked participants for feedback, many of whom described a positive experience that facilitated in‐depth reflection of the root causes of food insecurity in early childhood. An alternative facilitation strategy to motivate participants throughout the process could involve assessing their knowledge after each section, thereby demonstrating how their understanding of the system progresses over the GMB sessions. Second, a challenge in the process of building the CLD was to educate participants in a brief time on system thinking approaches and the expected outputs. Third, the model was based on a relatively small group of individuals with expertise in the topic or programs. Although families were not directly involved in the modeling sessions, their circumstances and perspectives were indirectly represented through the ongoing roles of specialists in program implementation and monitoring. However, we acknowledge that specialists may or may not have expertise in the cultures that influence families with young children. Additionally, during the interpretation of our CLD analysis, the research team incorporated insights from extensive in‐depth interviews with families experiencing food insecurity (Buccini et al. 2025; Dos Santos et al. 2023). Therefore, future research should intentionally include the voices of families with young children in the modeling process. Fourth, during the CLD data analysis, our research team grouped individual factors into higher‐level variables to facilitate the overall visualization of the systems identified and their inner workings. One challenge that emerged from this process was consolidating, defining, and naming variables while preserving the collective knowledge of the systems achieved by the specialists during the GMB sessions. We acknowledge that this process was subjective and that bias may have been introduced, reflecting specific knowledge that the research team had before and because of this study. To increase the transparency of this process and minimize bias from the research team, we included a positionality statement andSupporting materials documenting the grouping as well as clear operational definitions for each variable (Table 2). Lastly, classifying variables across NCF components posed some challenges. Our choice to categorize contextual factors (e.g., socioeconomic status) within NCF domains reflects an adaptation of the original framework, which may reduce comparability with studies that follow Black et al. (2017) more closely. On the other hand, this adaptation also aligns with evolving uses of the framework and offers a more comprehensive view of systemic influences (Skouteris et al. 2022; UNICEF, World Bank, & World Health Organization 2018). In conclusion, our study used an inclusive and participatory systems thinking approach suitable for generating insights that can be used to develop or strengthen equitable policies and programs. The process of co‐producing the CLD with a group of multisectoral specialists was a powerful way of eliciting, organizing, and integrating expert knowledge about the structure underlying a problematic trend. Our analysis supported the hypothesis that Brazil's pattern of food insecurity over the last 20 years was generated by an underlying system structure that contains the key components of the NCF. The analysis highlighted key feedback loops that were likely weakened in the latter half of the period due to political decisions and the COVID‐19 pandemic, which led to worsening food insecurity. Our findings indicate that a multi‐component package targeting households must be centered on the comprehensive NCF including nutrition, health, early education, social protection, and parenting education. Together these interventions targeting households and the community and regional level could strengthen the resilience of the system against future external shocks. Future research should test the CLD structure using system dynamic simulation models to evaluate the potential policy levers identified or through implementation research to determine how to apply and assess the model (or variations of it) to existing situations.

## Author Contributions

G.B. conceptualized the study, methodology and drafted the manuscript. K.A.S. and K.M. provided methodological expertise. G.B., K.A.S., K.M., M.G. conducted data analysis. M.G., S.I.V., R.P.E. contributed intellectually with review and editing versions of the manuscript draft.

## Conflicts of Interest

The authors declare no conflicts of interest.

## Supporting information


**Appendix 1:** Consolidated criteria for reporting qualitative research checklist (COREQ).

**Appendix 2:** Example of a script available at Scriptapedia website.

**Appendix 3:** Seed CLD.

**Appendix 4:** Relationships Among Variables Mapped in the Causal Loop Diagram of Early Childhood Food Insecurity in Brazil.

## Data Availability

The data that support the findings of this study are available from the corresponding author upon reasonable request.
