# Supplementary material for: Nurturing Care Systems Underlying Early Childhood Food Insecurity in Brazil: A Causal Loop Diagram Approach
Source: Matern Child Nutr. 2025 Nov 29;22(1):e70142. doi: 10.1111/mcn.70142 (PMC12663696; doi:10.1111/mcn.70142)
Supplement: Supplementary file 1 — Appendix 1: Consolidated criteria for reporting qualitative research checklist (COREQ). [file MCN-22-e70142-s004.docx]

**Appendix 1. Consolidated criteria for reporting qualitative research checklist (COREQ)**

A checklist of items that should be included in reports of qualitative research. Below is reported the section where authors considered each of the items listed in this checklist. A note N/A was included when the information was not relevant.

| **Topic** | **Item No.** | **Guide Questions/Description** | **Reported on Section** |
| --- | --- | --- | --- |
| **Domain 1: Research team and reflexivity** | | | |
| *Personal characteristics* | | | |
| Interviewer/facilitator | 1 | Which author/s conducted the interview or focus group? | Methods, Development of the CLD, Participants |
| Credentials | 2 | What were the researcher’s credentials? E.g. PhD, MD | Methods, Development of the CLD, Research Team Positionality |
| Occupation | 3 | What was their occupation at the time of the study? | Methods, Development of the CLD, Research Team Positionality |
| Gender | 4 | Was the researcher male or female? | N/A |
| Experience and  training | 5 | What experience or training did the researcher have? | Methods, Development of the CLD, Research Team Positionality |
| *Relationship with participants* | | | |
| Relationship  established | 6 | Was a relationship established prior to study commencement? | Methods, Development of the CLD, Research Team’s Positionality |
| Participant knowledge of the interviewer | 7 | What did the participants know about the researcher? e.g. personal goals, reasons for doing the research | Methods, Development of the CLD, Building the CLD |
| Interviewer  characteristics | 8 | What characteristics were reported about the interviewer/facilitator? e.g. Bias, assumptions, reasons and interests in the research topic | Methods, Development of the CLD, Research Team Positionality |
| **Domain 2: Study design** | | | |
| *Theoretical framework* | | | |
| Methodological  orientation and Theory | 9 | What methodological orientation was stated to underpin the study? e.g. grounded theory, discourse analysis, ethnography, phenomenology, content analysis | Methods, Analysis of the CLD |
| *Participant selection* | | | |
| Sampling | 10 | How were participants selected? e.g. purposive, convenience, consecutive, snowball | Methods, Development of the CLD, Participants |
| Method of approach | 11 | How were participants approached? e.g. face-to-face, telephone, mail, email | Methods, Development of the CLD, Sessions structure |
| Sample size | 12 | How many participants were in the study? | Methods, Development of the CLD, Participants |
| Non-participation | 13 | How many people refused to participate or dropped out? Reasons? | Methods, Development of the CLD, Participants |
| *Setting* | | | |
| Setting of data  collection | 14 | Where was the data collected? e.g. home, clinic, workplace | Methods, Development of the CLD, Sessions structure |
| Presence of non  participants | 15 | Was anyone else present besides the participants and researchers? | Methods, Development of the CLD, Sessions structure |
| Description of sample | 16 | What are the important characteristics of the sample? e.g. demographic data, date | Methods, Development of the CLD, Participants |
| *Data collection* | | | |
| Interview guide | 17 | Were questions, prompts, guides provided by the authors? Was it pilot tested? | Methods, Development of the CLD, Sessions structure |
| Repeat interviews | 18 | Were repeat inter views carried out? If yes, how many? | N/A |
| Audio/visual recording | 19 | Did the research use audio or visual recording to collect the data? | Methods, Development of the CLD, Sessions structure |
| Field notes | 20 | Were field notes made during and/or after the interview or focus group? | Methods, Development of the CLD, Sessions structure & Building the CLD |

Page 2 of 17

| Duration | 21 | What was the duration of the inter views or focus group? | Methods, Development of the CLD, Sessions structure |
| --- | --- | --- | --- |
| Data saturation | 22 | Was data saturation discussed? | Methods, Building the CLD |
| Transcripts returned | 23 | Were transcripts returned to participants for comment and/or correction? | Methods, Building the CLD |
| **Domain 3: analysis and findings** | | | |
| *Data analysis* | | | |
| Number of data coders | 24 | How many data coders coded the data? | Methods, Development of the CLD, Sessions structure |
| Description of the coding tree | 25 | Did authors provide a description of the coding tree? | N/A |
| Derivation of themes | 26 | Were themes identified in advance or derived from the data? | Methods, Development of the CLD, Sessions structure |
| Software | 27 | What software, if applicable, was used to manage the data? | N/A |
| Participant checking | 28 | Did participants provide feedback on the findings? | Methods, Building the CLD |
| *Reporting* | | | |
| Quotations presented | 29 | Were participant quotations presented to illustrate the themes/findings? Was each quotation identified? e.g. participant number | N/A |
| Data and findings  consistent | 30 | Was there consistency between the data presented and the findings? | Results |
| Clarity of major themes | 31 | Were major themes clearly presented in the findings? | Results |
| Clarity of minor themes | 32 | Is there a description of diverse cases or discussion of minor themes? | Results |

Developed from: Tong A, Sainsbury P, Craig J. Consolidated criteria for reporting qualitative research (COREQ): a 32-item checklist for interviews and focus groups. International Journal for Quality in Health Care. 2007. Volume 19, Number 6: pp. 349 – 357
