# Supplementary material for: Nurturing Care Systems Underlying Early Childhood Food Insecurity in Brazil: A Causal Loop Diagram Approach
Source: Matern Child Nutr. 2025 Nov 29;22(1):e70142. doi: 10.1111/mcn.70142 (PMC12663696; doi:10.1111/mcn.70142)
Supplement: Supplementary file 2 — Appendix 2: Example of a script available at Scriptapedia website. [file MCN-22-e70142-s001.docx]

| **Appendix 2. Example of a script available at Scriptapedia website.^[[1]](#footnote-1)^** |
| --- |
| **Concept Model:** This script is used at the start of a group model building project to introduce the process of modeling and symbolism of a model to participants. |
| **Status:** Best practices |
| **Primary nature of group task:** Presentation |
| **Time**  Preparation time: 180 minutes  Time required during session: 45 minutes  Follow-up time: 0 minutes |
| **Materials** |
| 1. White board or flip chart paper on easel with markers |
| 1. Computer and projector |
| **Inputs:** None |
| **Outputs** |
| - Familiarity with stock and flow diagrams and causal links |
| - Understanding that maps can be quantified and simulated |
| - Understanding that models can be created for the group's problem(s) |
| - Understanding that the model is owned by the group and can be repeatedly modified and improved |
| **Roles** |
| - [Modeler Facilitator](https://en.wikibooks.org/wiki/Scriptapedia/Roles_in_Group_Model_Building) to design the concept model |
| - [Modeler](https://en.wikibooks.org/wiki/Scriptapedia/Roles_in_Group_Model_Building) to show and run the formal model |
| **Steps** |
| 1. The Modeler Facilitator draws by hand the first version of the concept model on the white board. The modeler demonstrates/draws the tub with faucet and drain to explain stock & flow diagrams. |
| 1. The Modeler Facilitator then projects the first quantified version of the concept model from the computer. The first quantified version of the concept model is identical to the first version drawn on the white board. Next, the modeler simulates and traces the behavior produced by the model. |
| 1. The Modeler then elicits additional elements from participants adding one or more elements to the first version of the model on the white board to get an amended concept model (second version). Project the second version of the concept model from the computer. Simulate the second version of the concept model and trace its behavior over time. The behavior should be different, and the group should be told, “Behavior is a consequence of structure." |
| 1. Repeat step 3. |
| 1. The Modeler Facilitator summarizes the lessons as follows: the symbols that will be used, concept models can be quantified and simulated, behavior can be generated endogenously, changing structure changes behavior, maps and models can be repeatedly refined, and groups can own the models they create. |
| **Evaluation Criteria:** |
| - Participants are talkative, wanting to tell the modeler how the model is wrong and can be improved. |
| - Participants can use the symbols and terms of system dynamics to express their own experience and knowledge of systems. |
| **Authors** George P. Richardson |
| **History** First described by Richardson and Andersen (1995) and then published by Richardson (2013). |
| **Revisions** None |
| **References** |
| Richardson, G. P. and Andersen, D. F. (1995), Teamwork in group model building. *System Dynamics Review, 11*, 113–137. |
| Richardson, G. P. (2013). Concept models in group model building. *System Dynamics Review, 29*, 42-55. |

1. Scriptapedia. (n.d.-a). Causal Mapping in Small Groups. Available from: https://en.wikibooks.org/wiki/Scriptapedia/Causal_Mapping_in_Small_Groups [↑](#footnote-ref-1)
