# Supplementary figures and images for: Nurturing Care Systems Underlying Early Childhood Food Insecurity in Brazil: A Causal Loop Diagram Approach
Source: Matern Child Nutr. 2025 Nov 29;22(1):e70142. doi: 10.1111/mcn.70142 (PMC12663696; doi:10.1111/mcn.70142)

## Appendix 3, Seed CLD

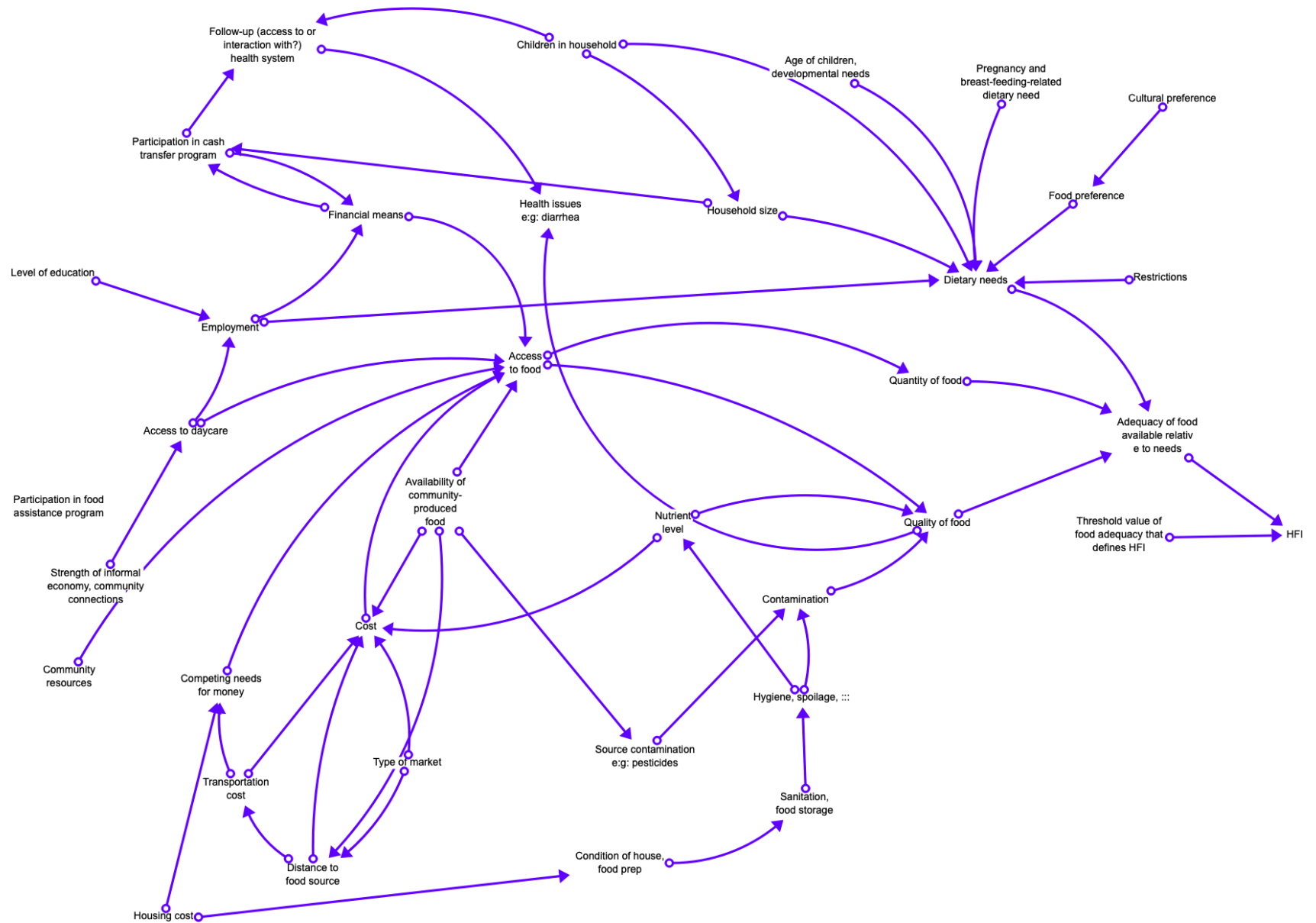

## Appendix 3, Complex CLD

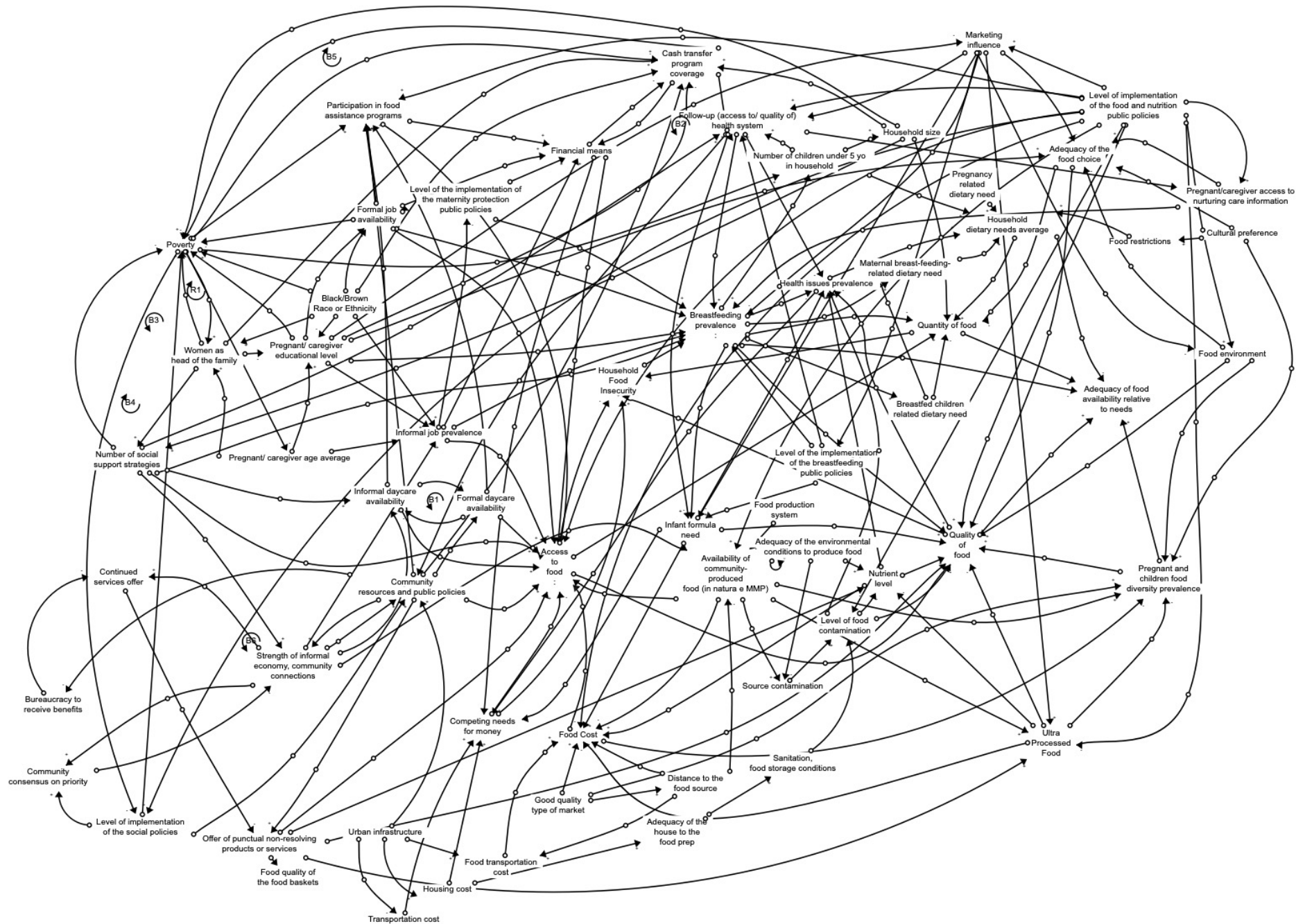

Supplement: Supplementary file 3 — Appendix 3: Seed CLD. [file MCN-22-e70142-s002.pdf]
