# Supplementary material for: Nurturing Care Systems Underlying Early Childhood Food Insecurity in Brazil: A Causal Loop Diagram Approach
Source: Matern Child Nutr. 2025 Nov 29;22(1):e70142. doi: 10.1111/mcn.70142 (PMC12663696; doi:10.1111/mcn.70142)
Supplement: Supplementary file 4 — Appendix 4: Relationships Among Variables Mapped in the Causal Loop Diagram of Early Childhood Food Insecurity in Brazil. [file MCN-22-e70142-s003.docx]

Appendix 3. Relationships Among Variables Mapped in the Causal Loop Diagram of Early Childhood Food Insecurity in Brazil.

| **Nurturing Care Domain** | **Variables** | **Variable affected by (outputs)** | **Polarity** | **Variable affects (inputs)** | **Polarity** |
| --- | --- | --- | --- | --- | --- |
| **Good Health** | Early Childhood Health & Development | Participation in Day Care | + |  |  |
|  |  | Participation in Health Services | + |  |  |
|  |  | Household Strain and Stress | - |  |  |
|  |  | Positive Parenting Practices | + |  |  |
|  |  | Early Childhood Food Insecurity | - |  |  |
|  | Participation in Health Services | Participation in Conditional Cash Transfer Program | + | Household Health Vulnerability | - |
|  |  |  |  | Household Food Literacy | + |
|  |  |  |  | Household Food Safety and Hygiene Practices | + |
|  | Household Health Vulnerability | Participation in Health Services | - |  |  |
| **Responsive Caregiving** | Participating in Positive parenting Education |  |  | Positive Parenting Practices | + |
|  | Positive Parenting Practices | Participating in Positive parenting Education | + | Early Childhood Food Insecurity | - |
|  |  | Household Strain and Stress | - |  |  |
| Opportunities for Early Learning | Household Strain and Stress | Food Insecurity of Households with Young Children | + | Early Childhood Health & Development | - |
|  |  | Quality of Food in Household | - | Positive Parenting Practices | - |
|  |  |  |  | Household Income | - |
|  |  |  |  | Household Food-related Coping Strategies | + |
|  | Participation in Day Care |  |  | Household Income | + |
|  |  |  |  | Early Childhood Health & Development | + |
|  |  |  |  | Early Childhood Food Insecurity | - |
| **Adequate Nutrition** | Quality of Food in Household | Household Food-related Coping Strategies | - | Household Strain and Stress | - |
|  |  | Household Food Safety and Hygiene Practices | + | Food Insecurity of Households with Young Child | - |
|  |  | Quality of Food Environment Available to Households | + |  |  |
|  |  | Healthy Food Cost | - |  |  |
|  |  | Ability to Make Healthy Food Choices when Purchasing Food | + |  |  |
|  |  | Physical Access to Healthy Food | + |  |  |
|  |  | Money Available to Buy Food | + |  |  |
|  | Quantity of Food in Household | Money Available to Buy Food | + | Food Insecurity of Households with Young Children | - |
|  |  | Household Food-related Coping Strategies | + |  |  |
|  |  | Quality of Food Environment Available to Households | + |  |  |
|  |  | Healthy Food Cost | - |  |  |
|  |  | Physical Access to Healthy Food | + |  |  |
|  | Ability to Make Healthy Food Choices when Purchasing Food | Money Available to Buy Food | + | Quality of Food in Household | + |
|  |  | Household Food Literacy | + |  |  |
|  |  | Quality of Food Environment Available to Households | + |  |  |
|  |  | Healthy Food Cost | - |  |  |
|  |  | Physical Access to Healthy Food | + |  |  |
|  | Household Food Literacy | Participation in Health Services | + | Ability to Make Healthy Food Choices when Purchasing Food | + |
|  | Physical Access to Healthy Food | Quality of Food Environment Available to Households | + | Quantity of Food in Household | + |
|  |  | Quality of Community Infrastructure | + | Quality of Food in Household | + |
|  |  |  |  | Ability to Make Healthy Food Choices when Purchasing Food | + |
|  | Healthy Food Cost | Quality of Food Environment Available to Households | - | Quantity of Food in Household | - |
|  |  |  |  | Quality of Food in Household | - |
|  |  |  |  | Ability to Make Healthy Food Choices when Purchasing Food | - |
|  | Quality of Food Environment Available to Households |  |  | Quantity of Food in Household | + |
|  |  |  |  | Quality of Food in Household | + |
|  |  |  |  | Ability to Make Healthy Food Choices when Purchasing Food | + |
|  |  |  |  | Healthy Food Cost | - |
|  |  |  |  | Physical Access to Healthy Food | + |
|  | Household Food Safety and Hygiene Practices | Participation in Health Services | + | Quality of Food in Household | + |
|  |  | Quality of Household Infrastructure | + |  |  |
|  | Household Food-related Coping Strategies | Money Available to Buy Food | - | Quality of Food in Household | - |
|  |  | Household Strain and Stress | + | Quantity of Food in Household | + |
|  |  | Food Insecurity of Households with Young Child | + |  |  |
|  | Household Nutritional Needs | Household Size | + | Food Insecurity of Households with Young Children | + |
|  | Nutritional Needs per Young Child |  |  | Early Childhood Food Insecurity | + |
| **Safety and Security** | Household Income | Participation in Conditional Cash Transfer Program | + | Household Socioeconomic Vulnerability | - |
|  |  | Participation in Day Care | + | Money Available to Buy Food | + |
|  |  | Household Socioeconomic Vulnerability | - | Quality of Household Infrastructure | + |
|  |  | Household Strain and Stress | - |  |  |
|  | Participation in Conditional Cash Transfer Program |  |  | Participation in Health Services | + |
|  |  |  |  | Household Income | + |
|  | Household Size |  |  | Household Nutritional Needs | + |
|  | Money Needed for Non-Food Expenses |  |  | Household Socioeconomic Vulnerability | + |
|  |  |  |  | Money Available to Buy Food | - |
|  | Money Available to Buy Food | Household Income | + | Household Food-related Coping Strategies | - |
|  |  | Money Needed for Non-Food Expenses | - | Quantity of Food in Household | + |
|  |  |  |  | Quality of Food in Household | + |
|  |  |  |  | Ability to Make Healthy Food Choices when Purchasing Food | + |
|  | Household Socioeconomic Vulnerability | Household Income | - | Household Income | - |
|  |  | Money Needed for Non-Food Expenses | + |  |  |
|  | Quality of Household Infrastructure | Household Income | + | Household Food Safety and Hygiene Practices | + |
|  |  | Quality of Community Infrastructure | + |  |  |
|  | Quality of Community Infrastructure |  |  | Quality of Household Infrastructure | + |
|  |  |  |  | Physical Access to Healthy Food | + |
| **Outcomes** | Food Insecurity of Households with Young Children | Household Nutritional Needs | + | Household Food-related Coping Strategies | + |
|  |  | Quantity of Food in Household | - | Early Childhood Food Insecurity | + |
|  |  | Quality of Food in Household | - | Household Strain and Stress | + |
|  | Early Childhood Food Insecurity | Participation in Day Care | + | Early Childhood Health & Development | - |
|  |  | Positive Parenting Practices | - |  |  |
|  |  | Food Insecurity of Households with Young Children | + |  |  |
|  |  | Nutritional Needs per Young Child | + |  |  |
